# Layer-by-layer Assembly of Nanosheets with Matching Size and Shape for More Stable Membrane Structure than Nanosheet-Polymer Assembly

**DOI:** 10.1021/acsami.4c03891

**Published:** 2024-05-08

**Authors:** Monong Wang, Young-Jin Song, Wenli Jiang, Francesco Fornasiero, Jeffrey J. Urban, Baoxia Mi

**Affiliations:** † Department of Civil and Environmental Engineering, University of California, Berkeley, California 94720, United States; ‡ Biosciences and Biotechnology Division, Lawrence Livermore National Laboratory, Livermore, California 94550, United States; § Molecular Foundry, Lawrence Berkeley National Laboratory, Berkeley, California 94720, United States

**Keywords:** two-dimensional materials, layer-by-layer assembly, MoS_2_ nanosheets, functionalized GO nanosheets, membrane swelling

## Abstract

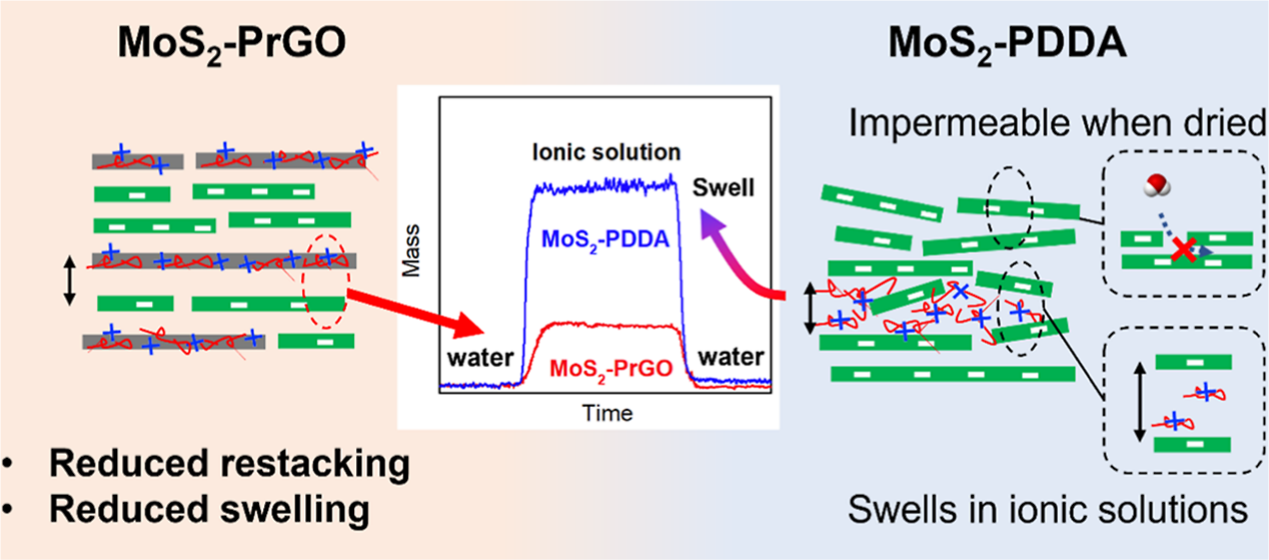

Layer-by-layer (LbL) assembly of oppositely charged materials has been widely used as an approach to make two-dimensional (2D) nanosheet-based membranes, which often involves 2D nanosheets being alternately deposited with polymer-based polyelectrolytes to obtain an electrostabilized nanosheet-polymer structure. In this study, we hypothesized that using 2D nanosheets with matching physical properties as both polyanions and polycations may result in a more ordered nanostructure with better stability than a nanosheet-polymer structure. To compare the differences between nanosheet–nanosheet vs nanosheet-polymer structures, we assembled negatively charged molybdenum disulfide nanosheets (MoS_2_) with either positively charged graphene oxide (PrGO) nanosheets or positively charged polymer (PDDA). Using combined measurements by ellipsometer and quartz crystal microbalance with dissipation, we discovered that the swelling of MoS_2_–PrGO in ionic solutions was 60% lower than that of MoS_2_–PDDA membranes. Meanwhile, the MoS_2_–PrGO membrane retained its permeability upon drying, whereas the permeability of MoS_2_–PDDA decreased by 40% due to the restacking of MoS_2_. Overall, the MoS_2_–PrGO membrane demonstrated a better filtration performance. Additionally, our X-ray photoelectron spectroscopy results and analysis on layer density revealed a clearer transition in material composition during the LbL synthesis of MoS_2_–PrGO membranes, and the X-ray diffraction pattern suggested its resemblance to an ordered, layer-stacked structure. In conclusion, the MoS_2_–PrGO membrane made with nanosheets with matching size, shape, and charge density exhibited a much more aligned stacking structure, resulting in reduced membrane swelling under high salinity solutions, controlled restacking, and improved separation performance.

## 1 Introduction

Emerging two-dimensional (2D) materials have shown their great potential as innovative membrane materials, addressing the pressing need for water reclamation and the treatment of unconventional water resources.^1^ Laminar membranes assembled from 2D materials such as graphene derivatives, zeolites, and transition metal dichalcogenides (TMDs) could yield fast and selective transport^2,3^ that overcomes the permeability-selectivity trade-off^4,5^ of current polymeric membranes. Among those rising 2D membrane materials, molybdenum disulfide (MoS_2_) as a representative TMD has shown its unique potential as a membrane filter with promising properties including high water flux, good heavy metal removal capability, and antifouling properties.^6−8^ Thus, integrating MoS_2_ into existing membrane technologies, such as ultrafiltration (UF) or nanofiltration (NF), could offer an effective approach to creating high-performance membranes.

While 2D MoS_2_ membranes possess numerous benefits, the membrane synthesis technique needs to be carefully engineered to reach the membrane’s full potential. Layer-stacked membranes made by vacuum filtration have been widely used to study membrane properties. However, it was discovered that the MoS_2_ layers would restack and the membrane would irreversibly lose its permeability as well as compromise its heavy metal removal capabilities once dried.^7^ To solve this problem, intercalating molecules between the stacked MoS_2_ layers by methods including surface functionalization^9^ and cation intercalation^10^ has been shown to be effective. Alternatively, the layer-by-layer (LbL) assembly technique can also be implemented as a simple, cost-effective, and scalable solution that can reduce the restacking of MoS_2_ without tedious material modifications.^11,12^

LbL membranes are generally assembled by alternately depositing oppositely charged constituents, which are held together through electrostatic forces. With the negative surface charge of MoS_2_, a variety of polycations can be selected for the synthesis of LbL layers and those polycations naturally become spacers between MoS_2_ layers. While polymer-based polycations are commonly used for LbL assembly,^13−15^ the excellent performance of nanocomposites with 2D materials being the major component^16^ suggests that nanosheet-based polycations could offer significant benefits in the synthesis of LbL membranes. Nanosheet-based polycations not only offer intrinsic properties such as durability and antifouling characteristics^17,18^ but also have the potential to yield high water permeability with good selectivity due to their structural similarity to layer-stacked membranes. However, a comprehensive study on the difference between polymer-based and nanosheet-based LbL membranes has not yet been performed.

To evaluate the differences between nanosheet–nanosheet assembled LbL membranes and nanosheet-polymer membranes, we synthesized one membrane by assembling negatively charged MoS_2_ nanosheets and positively charged rGO (PrGO) nanosheets, and the other membrane by assembling MoS_2_ nanosheets with the PDDA polymer (Poly diallyldimethylammonium chloride). Quartz crystal microbalance with dissipation (QCM-D) was used to quantitatively analyze the composition of PrGO/PDDA and MoS_2_ in the membranes, as well as the membrane swelling behavior in solutions under different ionic strengths and pH conditions. The chemical composition, interlayer spacing, and filtration performance of the membranes were also closely examined and compared. Our results indicate that the nanosheet–nanosheet membrane is more stable and has better resistance to swelling and restacking than the nanosheet-polymer membrane.

## 2 Materials and Methods

### 2.1 Synthesis and Characterization of PDDA-Functionalized GO (PrGO) and MoS_2_ Nanosheets

PrGO nanosheets were synthesized by functionalizing reduced GO nanosheets with poly diallyldimethylammonium chloride (PDDA) using a modified procedure from Zhang et al.^19^ PDDA is a long chain polymer with high positive charge density from its quaternary amine groups, and it is widely used for nanocomposite synthesis and water treatment. The attachment of PDDA on GO nanosheets is most likely by π–π interactions between the unsaturated impurity in PDDA and the basal plane of GO.^20,21^ The GO nanosheets were mildly reduced to both improve the loading of PDDA and reduce the negative charge density generated by the oxygenated groups in GO, therefore ensuring an overall positive charge on the nanosheets. To synthesize the PrGO nanosheets, a GO suspension with a concentration of 4 g/L was first prepared using the modified Hummer’s method^13^ (see Text S1 for detailed procedures). 100 mg of GO was diluted to 250 mL with Milli-Q grade water, followed by 15 min bath sonication. 4 mL of 30% ammonia solution, 3 mL of 35% hydrazine solution, and 0.25 mL of 20% PDDA (medium molecular weight, 200–350 kDa) was then added to the GO suspension under mild stirring at 250 rpm. Note that the quantity of hydrazine and ammonia can be altered to modify the GO reduction state. Aggregation of GO was observed once PDDA is added to the suspension. The suspension was then continuously mixed for 1 h under 93 °C heating and quickly cooled down to room temperature with an ice bath to terminate the reaction. Then the suspension was washed with ethanol and water using centrifugation at 8000 rpm at least 2 times each to remove the unbounded PDDA. The solid residue was then collected and diluted to the desired concentration. To break down aggregated nanosheets, the suspension was sonicated using a probe sonicator (Q500, Qsonica, CT) at 50% intensity for 30 min with an ice bath. Finally, the suspension was subjected to centrifugation again at 8000 rpm to remove any nanosheets remaining aggregated, and the supernatant was collected for future tests. Milli-Q water (Smart2Pure water system, Fisher Scientific, Waltham, MA) was the default water used in this study and has a conductivity of around 1.7 μS/cm. All chemicals were purchased from Sigma-Aldrich (St. Louis, MO) unless otherwise stated.

MoS_2_ nanosheets were prepared using chemical exfoliation.^22^ 3 mL of 1.6 M *n*-butyllithium hexane solution was added to 300 mg of MoS_2_ powder (<2 μm) under moderate stirring for 2 days. The lithium-intercalated MoS_2_ was washed with hexane to remove excess reagents and organic byproducts. MQ was immediately added to MoS_2_ after the hexane wash to exfoliate bulk MoS_2_ into nanosheets. The suspension was then subjected to bath sonication for 1 h, then transferred to dialysis in water overnight with nitrogen purging to remove LiOH and organic byproducts. Finally, the suspension was sonicated for 5 min using bath sonication and centrifuged at 3000 rpm for 20 min to remove the unexfoliated MoS_2_.

The size and charge of the nanosheet suspensions were characterized by a Zetasizer Nano ZS90 (Malvern Instruments, UK). The shape and thickness of the nanosheets were characterized using atomic force microscopy (AFM, Cypher ES, Oxford Instruments, MA). The elemental composition and interlayer spacing of layer-stacked nanosheets were characterized using X-ray photoelectron spectroscopy (K-Alpha X-ray Photoelectron Spectrometer (XPS) System, Thermo Fisher Scientific, Waltham, MA) and an X-ray diffractometer (XRD, Bruker, Madison, WI), respectively. Layer-stacked nanosheet samples were prepared by drop casting nanosheet suspensions on silica wafers. Samples were dried under vacuum overnight before test.

The concentration of the nanosheet suspension was determined using the QCM-D (E-1, Biolin, Sweden).^23^ In short, 0.05 mL of nanosheet suspension was first deposited on a poly(ether sulfone) membrane (0.03 μm pore size, 47 mm diameter, Sterlitech, Auburn, WA, USA) using vacuum filtration with 47/35 mm glass frit. The nanosheet layer on the membrane was transplanted to a gold QCM-D sensor surface (1 cm^2^, Biolin Scientific, Linthicum Heights, MD, USA) and dried in the oven. The nanosheet mass was then measured using QCM-D. The material concentration was calculated by multiplying the measured mass with the total coated area of the membrane (9.62 cm^2^), and then divided by the added volume, 0.05 mL. To calculate the density of layer-stacked nanosheets, ellipsometry measurements were performed to obtain the layer thickness using ellipsometer (FS-1 Multi-wavelength, Film Sense, Lincoln, NE, USA), and the density was calculated by dividing the QCM-D measured mass with thickness. To obtain the density of hydrated nanosheet, the transplanted nanosheet layer was kept hydrated before QCM-D and ellipsometry measurements.

### 2.2 LbL Membrane Fabrication and Characterization

A negatively charged membrane substrate was prepared using blended polysulfone/sulfonated polysulfone (sPSF) for LbL assembly.^21^ We first prepared a polymer solution by sequentially dissolving 10 g of polyvinylpyrrolidone, 6 g of sPSF (provided by BASF, Germany), and 18 g of PSF in 80 mL of *N*-methyl-2-pyrrolidone. The polymer solution was stirred for 4 h and degassed under vacuum overnight. Then the solution was cast on a clean glass plate using a casting rod (250 μm coating thickness) and submerged in pure water immediately for phase inversion. The membrane was then soaked in water for at least 1 week with frequent water changes to remove residual organic solvent.

To fabricate the MoS_2_–PrGO and MoS_2_–PDDA LbL membranes, the negatively charged sPSF membrane substrate was first soaked in presonicated polycation solutions (i.e., PrGO or PDDA) for 15 min followed by water rinsing to remove the excessive polycations to obtain a half bilayer (0.5 bilayer). Then the membrane was subsequently soaked in presonicated MoS_2_ suspensions for another 15 min, followed again with water rinsing to complete the bilayer. The above process was repeated for a specified number of cycles to fabricate the MoS_2_–PrGO and MoS_2_–PDDA membranes with the desired number of bilayers. The concentration of all polycation and MoS_2_ solutions used in the LbL process was 0.5 g/L, and the pH was controlled at around 7 by using hydrochloric acid (HCl) or sodium hydroxide (NaOH).

The stacking structure of the LbL membrane was characterized using XRD. Because the synthesized LbL membranes were too thin to get detectable intensity from XRD, free-standing LbL films were isolated from the sPSF polymer substrate and stacked together to obtain a thicker film for XRD characterization (see Text S2 for a detailed sample preparation method). The chemical state and elemental composition of the membranes after each layer deposition step (half and complete bilayers) were examined using XPS. The atomic ratio between quaternary amine N and Mo(IV), was calculated using the peak area and the relative structural factor of Mo 3p (11.83) and N 1s (1.68). The structural factors were obtained from the Avantage data system (Thermo Fisher Scientific, Waltham, MA). The overall elemental composition of the LbL membranes were also analyzed using the Avantage data system. The surface charge of LbL membranes and sPSF substrate was characterized using Zetasizer Nano ZS90 (Malvern Instruments, UK) with a surface zeta potential cell kit. The surface morphology of LbL membranes was characterized by scanning electron microscopy (SEM, Zeiss Gemini Supra 55, NY).

The process of LbL assembly was monitored using QCM-D along with an ellipsometer to study the mass and thickness change during LbL deposition, respectively. Two replicates were performed for all QCM-D and ellipsometry tests. The detailed characterization steps can be found in Text S3. To compare the amount of MoS_2_ deposited in MoS_2_–PrGO and MoS_2_–PDDA membranes, cumulative MoS_2_ mass content was calculated by
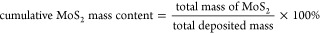
1

The density of the bilayers was calculated by
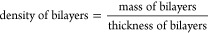
2

The loading of PDDA in MoS_2_–PrGO membrane was calculated by
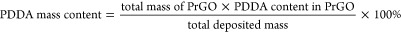
3

The swelling of the bilayers was also monitored using combined QCM-D and ellipsometry. Similar to the LbL assembly process, salt solutions including sodium chloride (NaCl), sodium sulfate (Na_2_SO_4_), and magnesium chloride (MgCl_2_) with ionic strength of 7.5, 15, 37.5, 75, 150, 375, and 750 mM were pumped into the QCM-D module. The pH of all testing solutions was controlled at around 7 using HCl or sodium hydroxide NaOH. Solutions with pH 2, 7, and 10 were also used to examine the effect of pH on membrane swelling. The ionic strengths for different pH conditions were adjusted to around 10 mM using NaCl. The mass and thickness were recorded for analysis when the swelling reached equilibrium. The swelling extent in each testing solution was then calculated by
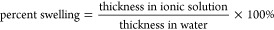
4

### 2.3 Membrane Filtration Performance Test

The filtration performance of the LbL membranes was tested using an Amicon stirred cell with a 10 mL volume (MiliporeSigma, MA). The filtration cell was operated under dead-end mode, and a pressurized water tank was connected to the cell to provide continuous feed solution. LbL membranes with 9 bilayers were selected for performance tests. Three replicates were performed for all of the filtration tests. The LbL membranes were precompressed at 60 psi (4.1 bar) and then tested under 40 psi (2.8 bar). The permeability was calculated by converting the rate of mass change to volume change using the density of water at 25 °C (0.997 g/cm^3^), then dividing by the operating pressure and membrane area (3.87 cm^2^). The unit of permeability was converted to liter/m^2^/h/bar (LMH/Bar). The rejection *R* was calculated by *R* = (1 – *C*_p_/*C*_R_) × 100%, where *C*_p_ and *C*_R_ are the concentrations of testing chemicals in the permeate and retentate solutions, respectively. The retentate and permeate were sampled and measured every hour for at least 3 h or until there was no change in rejection between three consecutive data points. This approach was taken to eliminate the potential impact of membrane adsorption.

300 mg/L polyethylene glycol (PEG) solutions (300 mg/L) with molecular weights (MW) of 200, 600, 1000, 2000, 3350, and 6000 Da were used to determine the membrane’s molecular weight cutoff (MWCO). The MWCO was defined as the lowest molecular weight of PEG in which 90% of the PEG was rejected by the membrane. The PEG concentration with a molecular weight below 1000 Da was determined using a total organic analyzer (TOC-L, Shimadzu, Japan). The PEG concentration with molecular weight higher than 1000 Da was determined using Dragendorff method^24^ (see Text S4 for detailed method description). 40 mg/L of positively charged Victoria B (VB) and negatively charged rhodamine WT (RWT) were used for membrane rejection tests. The concentration of VB and RWT were determined at absorption wavelengths of 616 and 554 nm, respectively, using a UV–vis spectrophotometer (UV160U, Shimadzu Scientific Instruments, Columbia, MD). The pH of all testing solutions was controlled at around 7 by adding HCl or NaOH.

## 3 Results and Discussion

### 3.1 Characterization of PrGO and MoS_2_ Nanosheets

**Figure 1:**
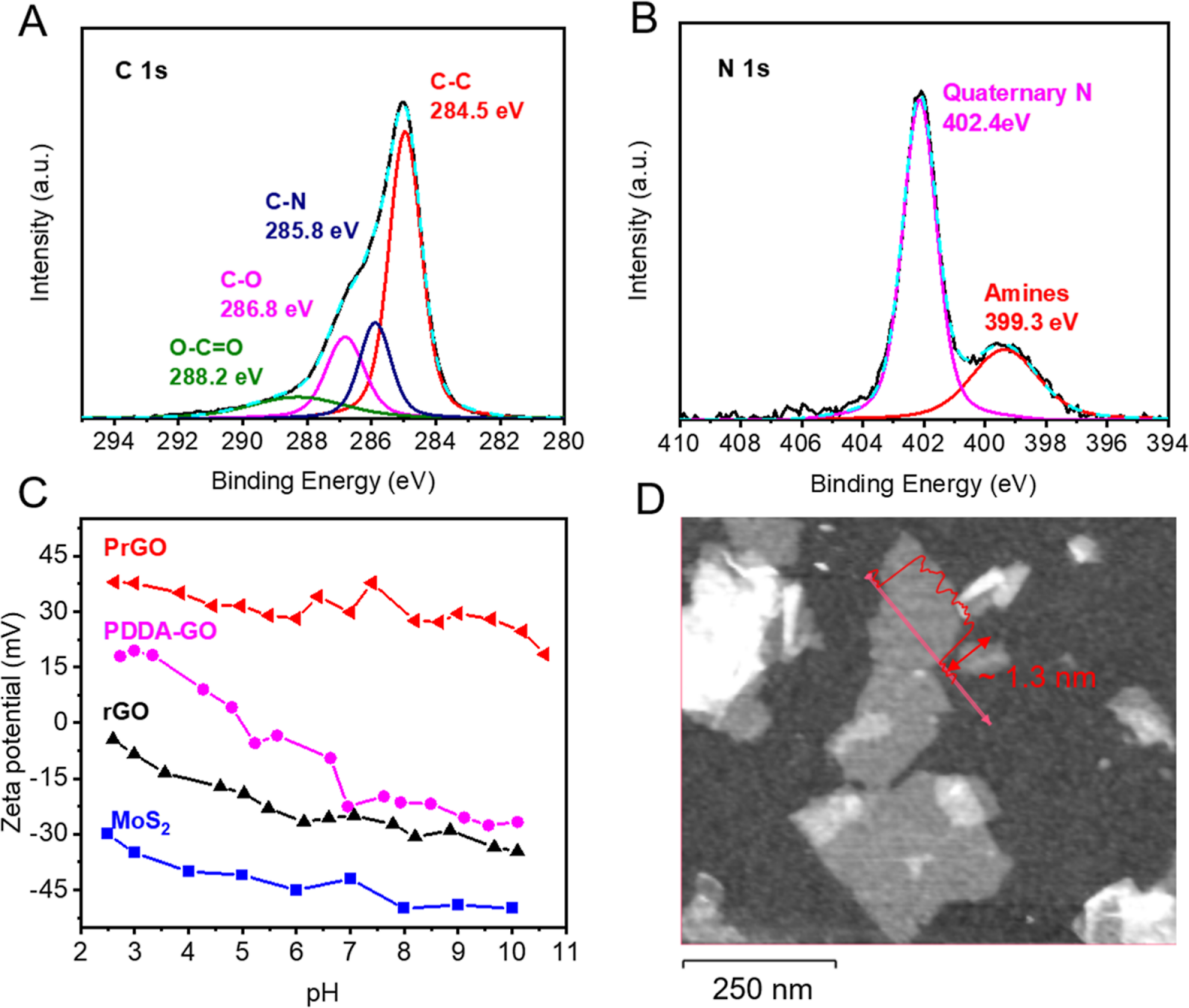
Characterization of the PrGO and MoS_2_ nanosheets. The XPS spectra of PrGO for C 1s (A) and N 1s (B). (C) The zeta potential of PrGO, PDDA–GO, rGO, and MoS_2_ suspensions at pH ranging between 2 and 10. (D) The AFM image of PrGO nanosheets with a profile indicating the thickness of PrGO, which is around 1.3 nm. The PrGO is PDDA-functionalized rGO nanosheets, and PDDA–GO is PDDA-functionalized GO nanosheets without reduction.

To confirm the success of functionalization, the chemical composition of PrGO was examined by using XPS. The presence of the C–N peak at 285.8 eV in Figure 1A and the quaternary amine peak at 402.4 eV in Figure 1B demonstrates that the PDDA was successfully attached to the rGO nanosheets.^19^ In comparison, reduced GO without PDDA functionalization did not have this quaternary amine peak (Figure S1A), but only the peak that belongs to the N–H bond at around 399.3 eV, which is likely caused by the hydrazine reduction process.^25^ Meanwhile, the reduction of GO can be observed from the C 1s spectra, where the peak intensity of the C–O (286.8 eV) and C=O (288.2 eV) bond in PrGO was much lower than that of the C–C bond (284.5 eV). In comparison, the PDDA-functionalized GO without any reduction step (PDDA–GO, Figure S1B) showed higher C–O and C=O intensity. The mass percentage of the functionalized PDDA on PrGO was estimated to be around 32% by analyzing the elemental composition of PrGO, obtained from XPS analysis (Figure S1C). Detailed calculations are available in the Supporting Information (Text S5).

The charge properties of PrGO were examined using a Zetasizer. As shown in Figure 1C, the PrGO had a strong positive charge at around 30 mV under pH range of 2–10. There was no charge reversal observed at any of the tested pH levels, indicating that the PrGO can establish a strong and stable electrostatic interaction with MoS_2_ over a wide pH range. In comparison, the PDDA–GO (without the GO reduction step) exhibited a charge reversal from positive to negative at pH 5, demonstrating the importance of the reduction step for maintaining a consistent positive charge for the nanosheets. The charge intensity, however, will not be further improved by increasing the reduction extent of rGO using 2 times higher concentration of hydrazine and ammonia during synthesis (Figure S2A,B, no significant difference on zeta potential was observed between strongly reduced and mildly reduced PrGO). This indicates that mild GO reduction conditions were sufficient to remove negatively charged functional groups and create PrGO nanosheets with a consistent positive charge under wide pH conditions.

To verify that the PrGO was indeed nanosheets and not aggregated particles, AFM was used to examine the thickness of PrGO. As shown in Figure 1D, the PrGO appeared to be nanosheets with a thickness around 1.3 nm and lateral length of 200–400 nm. This thickness was found to be higher than GO (which are typically around 0.8 nm)^26^ possibly due to the attached PDDA chains and/or inaccuracy of the AFM measurement. The average size of PrGO was determined to be around 300 nm using a Zetasizer, which was also confirmed by SEM imaging (Figure S2C,D). The density of dry PrGO determined by QCM-D and ellipsometry was around 2 g/cm^3^, slightly higher than the reported rGO density in other studies (1.5–1.9 g/cm^3^).^27^

The physicochemical properties of chemically exfoliated MoS_2_ nanosheets have been reported in our previous studies but were also confirmed here.^22^ For example, the XPS measurements showed that the MoS_2_ nanosheets have mixed 1T and 2H phases (Figure S3) that match those of previous reports. The zeta potential measurements in Figure 1C showed that the MoS_2_ nanosheet had a strong negative charge in the pH range of 2–10, reaching around −45 mV at neutral pH. AFM measurements (Figure S4A) revealed that the nanosheets have a thickness around 1 nm with lateral length of 200–500 nm that agrees well with previous reports.^28^ The AFM result was also consistent with the size measurement by Zetasizer, which showed an average size of 400 nm for the MoS_2_ suspensions (Figure S4B). The density of restacked MoS_2_ nanosheets after drying was around 3.5 g/cm^3^, determined by using QCM-D and ellipsometry measurements. This value is lower than the single crystal MoS_2_ density (5.06 g/cm^3^) due to enlarged interlayer spacing and defects from layer stacking.^29^

Overall, the PrGO and MoS_2_ nanosheets exhibited resemblance in their physical properties except for opposite charges, and thus we anticipated an ordered stacking when they are assembled into a MoS_2_–PrGO membrane, which would potentially improve membrane stability and filtration performance.

### 3.2 Synthesis of the MoS_2_–PrGO and MoS_2_–PDDA Membranes

**Figure 2:**
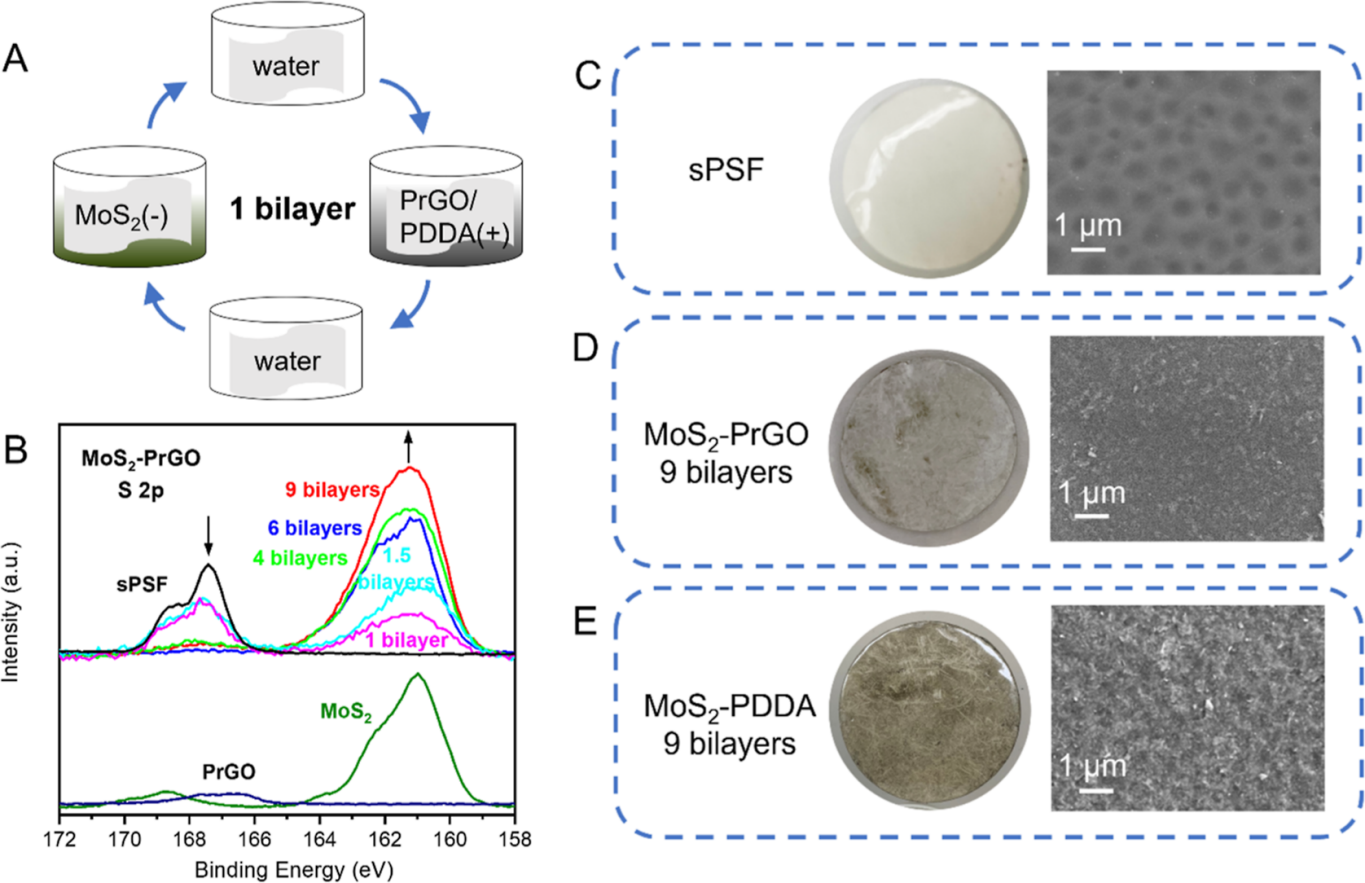
LbL assembly of MoS_2_–PrGO and MoS_2_–PDDA membranes. (A) Schematic illustration of the LbL assembly. 0.5 g/L MoS_2_, PrGO, and PDDA with solution pH at 7 was used in this study. (B) XPS of the MoS_2_–PrGO membrane with different bilayers. The peak of sPSF at 167.5 eV disappeared after 4 bilayers. The optical and SEM images of the white sPSF substrate (C), the gray/brownish MoS_2_–PrGO membrane (D), and the brownish MoS_2_–PDDA membrane (E). The SEM images show that the MoS_2_–PDDA membrane has a rougher surface than that of the MoS_2_–PrGO membrane.

MoS_2_–PrGO and MoS_2_–PDDA membranes were synthesized by LbL assembly of MoS_2_ and PrGO nanosheets or MoS_2_ and PDDA polymer (Figure 2A). Upon the deposition of bilayers, the membrane turned from white to gray-brown, and the color became darker with more deposition cycles (Figure S5). To examine whether the bilayers have fully covered the substrate, XPS analysis was performed on the LbL membranes (Figures 2B and S6). The S 2p peak from sPSF at 167.5 eV disappeared after 4 bilayers of deposition, indicating a complete coverage of the sPSF by the bilayers. Figure S7 demonstrates the Mo 3d scan for the LbL membranes. Compared to freshly prepared MoS_2_ (Figure S3), the atomic ratio of 1T only decreased by around 3% (which could be caused by MoS_2_ oxidation during LbL synthesis), therefore it is safe to say that the properties of MoS_2_ did not change during LbL synthesis. Figure 2C–E presents the photo and SEM images of the pristine and LbL membranes. The MoS_2_–PrGO appeared to be grayer than MoS_2_–PDDA, due to the intrinsic black color of PrGO. Meanwhile, the MoS_2_–PDDA membrane was visually darker, indicating a potentially higher concentration of deposited MoS_2_ nanosheets compared to the MoS_2_–PrGO membranes. Additionally, the SEM image shows that the surface morphology of MoS_2_–PDDA is rougher than that of MoS_2_–PrGO, possibly due to the use of PDDA that had caused aggregation of MoS_2_ during LbL synthesis. This aggregation can lead to the creation of more defects across the membranes, which could negatively impact membrane performances.^30^

### 3.3 Understand the Internal Stacking Structure of MoS_2_–PrGO and MoS_2_–PDDA Membranes

**Figure 3:**
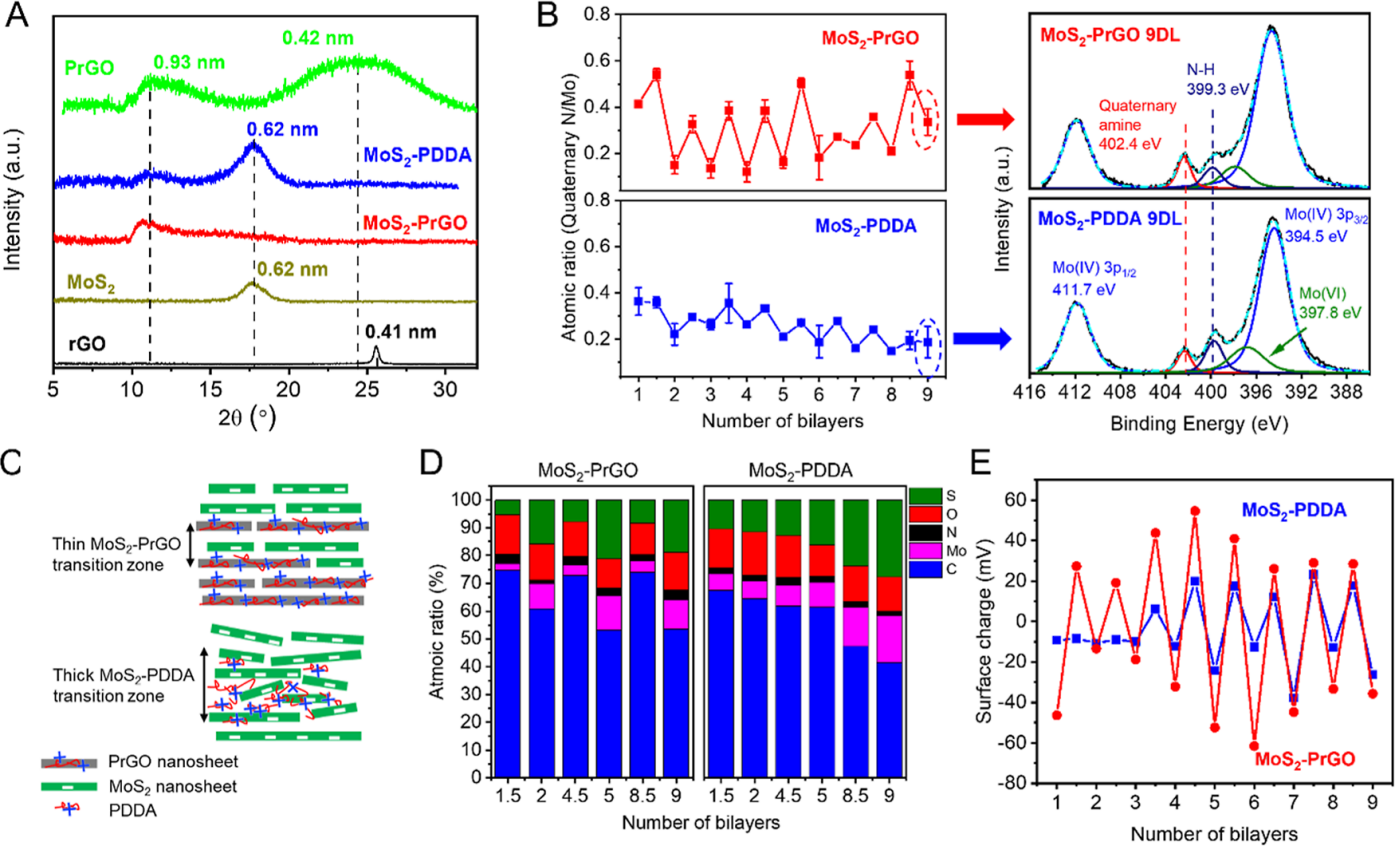
XRD, XPS spectroscopy, and charge characterization for MoS_2_–PrGO and MoS_2_–PDDA membranes. (A) XRD measurement for MoS_2_–PrGO and MoS_2_–PDDA membrane (isolated from the sPSF substrate), and PrGO, MoS_2_, and rGO nanosheets (drop cast). The samples were dried in an oven before test. (B) On the left: the atomic ratio between N from quaternary amine and Mo(IV), calculated using the peak area and the relative structural factor of Mo 3p (11.83) and N 1s (1.68); on the right: the XPS N 1s scan for 9-bilayer MoS_2_–PrGO membrane and 9-bilayer MoS_2_–PDDA membrane. The quaternary amine peak from PDDA is located at 402.4 eV, and the Mo(IV) 3p peaks from MoS_2_ are located at 394.5 eV (Mo 3p_3/2_) and 411.7 eV (Mo 3p_1/2_). (C) Schematic of the membrane bilayer structures, illustrating a thinner transition zone in the MoS_2_–PrGO than in the MoS_2_–PDDA membrane. (D) The atomic composition of the MoS_2_–PrGO and MoS_2_–PDDA membranes. (E) The membrane surface charge measured after depositing each layer during LbL synthesis.

To understand the internal stacking structure of MoS_2_–PrGO and MoS_2_–PDDA, the membranes were isolated from sPSF and transferred onto silica wafers for XRD characterization. The interlayer spacing of pure rGO, PrGO, and MoS_2_ membranes was also measured with XRD to obtain baseline structural properties. As shown in Figure 3A, two interlayer spacings were observed for pure PrGO, with 0.93 nm at a 2θ of 11° and 0.42 nm at a 2θ of 24.5°. The peak at 0.93 nm spacing was most likely attributed to the stacking of PrGO nanosheets.^31^ The broader peak at 0.42 nm could be due to the uneven distribution of PDDA on the PrGO nanosheets that would result in regions of rGO–rGO stacking, with an interlayer spacing close to that of a mildly reduced rGO (around 0.41 nm at 2θ of 25.2°).^32^ Note that other factors such as material strain and the small crystal size of the PrGO may also contribute to the broadened peak. The pure MoS_2_ demonstrated a single peak at around 17.5° representing 0.62 nm interlayer spacing, agreeing well with the dried structure of MoS_2_ from our previous study.^2^

The XRD results (Figure 3A) for the MoS_2_–PrGO membrane exhibited one broad peak at 11°, corresponding to an interlayer spacing of 0.93 nm. This peak is at the same position as that of layer-stacked PrGO, suggesting that the MoS_2_–PrGO stacking resembles layer-stacked membranes. Like our previous discussion on PrGO, the broadening of the peak could be due to the uneven distribution of PDDA on the PrGO nanosheets (i.e., regions free of PDDA on PrGO will have a smaller gap with MoS_2_ than regions covered by PDDA, resulting in an XRD signal at larger 2θ). On the other hand, two peaks were observed for the MoS_2_–PDDA membrane, including the typical peak of restacked MoS_2_ at 0.62 nm and the peak at similar position as the MoS_2_–PrGO at 0.93 nm. This indicated that while PDDA was intercalated into MoS_2_ layers during LbL deposition and formed a larger interlayer spacing, regions of aggregated MoS_2_ were also formed in MoS_2_–PDDA membranes.

The internal elemental composition of the membrane was characterized by XPS analysis. Membranes after each LbL deposition cycle were sampled for XPS scan, and the collected spectrum were used to calculate the relative atomic ratio of MoS_2_ to PDDA to obtain the material composition of each layer. Note that because XPS is a surface technique with a penetration depth of 1–10 nm, the collected spectrum should be mainly from the top layer of the membranes. The XPS N 1s spectra of a 9-bilayer MoS_2_–PrGO and a MoS_2_–PDDA membrane are shown in Figure 3B (graphs on the right) as an example. The presence of both N 1s and Mo 3p peaks in the spectrum suggested that a transition zone, a region where MoS_2_ and polycation (PrGO or PDDA) are mixed, exists between adjacent LbL layers. Note that this interdigitation between polycation and polyanion has long been observed in polymer-based LbL structures.^33,34^ We then calculated the atomic ratio between quaternary amine (representing deposited PDDA) and Mo (representing deposited MoS_2_) using the peak area and the relative structural factor of N 1s and Mo 3p peaks, and the results for each layer during LbL synthesis are shown in Figure 3B (graphs on the left). Compared to MoS_2_–PDDA, the MoS_2_–PrGO membrane exhibits a more drastic switch in the atomic ratio between Mo and the quaternary amine. It is possible that the MoS_2_–PDDA membrane has a thicker transition zone (i.e., mixed MoS_2_–PDDA region) at the bilayer interface than the MoS_2_–PrGO membrane, resulting in less change of N to Mo ratio between adjacent layers, as illustrated in Figure 3C.

Figure 3D shows the complete elemental composition change during the layer deposition. Similar to Figure 3B, a change in composition was observed between adjacent layers. For both MoS_2_–PDDA and MoS_2_–PrGO membranes, at half bilayer the carbon and nitrogen content were higher due to the exposed PrGO and PDDA and at full bilayer the sulfur and molybdenum content were higher due to the exposed MoS_2_. This change in elemental composition between adjacent layers was more apparent in MoS_2_–PrGO membranes. For example, in the 4.5th bilayer of MoS_2_–PrGO, the Mo content was 3.8% and the S content was 7.8%, while in the 5th bilayer, the Mo and S content increased to 12.3 and 21.9%, respectively. However, for MoS_2_–PDDA, in the 4.5th bilayer, the Mo and S content was 7.5 and 12.7%, while in the 5th bilayer, it was 9.1 and 16.3%, only increasing for around 3%. The C content also varied with the Mo content. In MoS_2_–PrGO, the C content varied by 15–20% between adjacent layers, while in MoS_2_–PDDA, the C content only varied by around 2–5%. Based on our discussion for Figure 3B, because the MoS_2_–PDDA membrane has a thicker transition zone, a significant portion of MoS_2_ had mixed with PDDA during layer deposition, resulting in less change in elemental composition between adjacent layers. This observation was further supported by the surface charge measurement after each layer deposition (Figure 3E), where the overall surface charge density within MoS_2_–PrGO layers was higher than that within MoS_2_–PDDA layers.

### 3.4 Quantify the Material Loading and Membrane Structure Change during LbL Synthesis Using Combined QCM-D and Ellipsometry Approach

**Figure 4:**
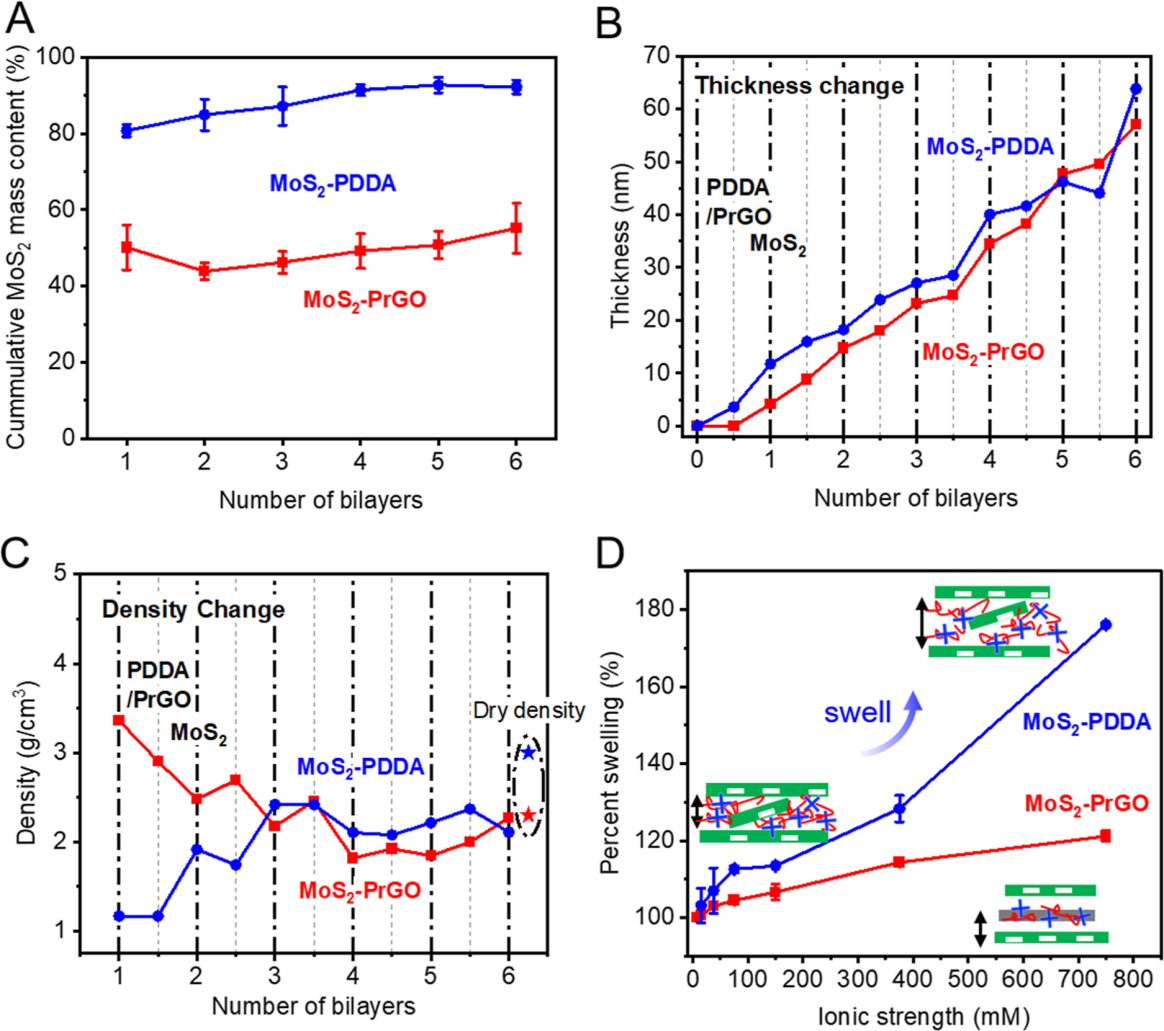
QCM-D and ellipsometry measurements of LbL assembled MoS_2_–PDDA and MoS_2_–PrGO membranes. (A) The cumulative mass content of MoS_2_. After depositing 6 bilayers, 90% of the mass in MoS_2_–PDDA membranes was MoS_2_, while in MoS_2_–PrGO, only 50% of the mass was MoS_2_. (B) The thickness and (C) the density change in the LbL membranes during bilayer deposition. (D) Swelling of MoS_2_–PrGO and MoS_2_–PDDA layers under ionic strength of 7.5, 15, 37.5, 75, 150, 375, and 750 mM, adjusted by Na_2_SO_4_.

To accurately quantify the MoS_2_ loading, QCM-D was used to monitor the mass change during LbL synthesis (Figure S8). The cumulative mass content of MoS_2_ during deposition was calculated using eq 1. The LbL membranes are fully hydrated during measurement. As shown in Figure 4A, during the deposition of the MoS_2_–PDDA membrane, the mass content of MoS_2_ gradually increased from 80% with more deposited bilayers, ultimately plateauing at 90%. In contrast, in the MoS_2_–PrGO membranes, the MoS_2_ only accounts for approximately 50% of the total mass throughout the deposition process. Although a slight increase of the MoS_2_ proportion was observed with more bilayers, the change was much smaller than that in the MoS_2_–PDDA membranes.

The observation of those two types of LbL membranes having different MoS_2_ proportions can be attributed to the different mobilities of PDDA used for synthesis. The PDDA molecules are hydrophilic due to their high charge density and have a high radius of gyration when dissolved in water (around 50 nm with *M*_w_ = 200 kDa).^35^ When synthesizing the MoS_2_–PDDA membranes with pure PDDA solution, the polymer chains of PDDA are free to rotate and deform which can anchor more MoS_2_ during synthesis. Contrarily, the movement of PDDA on the PrGO surface is more restrained and, subsequently, limits the deposition of MoS_2_. Note that the PDDA loading in MoS_2_–PrGO membranes is similar to or even higher than that in MoS_2_–PDDA. According to the elemental analysis of PrGO and PDDA with XPS in Section 3.1, PDDA accounts for 32% of PrGO, which results in 14–18% loading of PDDA in MoS_2_–PrGO bilayers. In MoS_2_–PDDA bilayers, the loading was 7–19% (Figure S9).

The mobility of PDDA can also be used to explain why previous characterizations suggested that MoS_2_–PDDA and MoS_2_–PrGO membranes have different structures. During the synthesis of MoS_2_–PDDA layers, the freeform PDDA caused more mixing between MoS_2_ and PDDA at the bilayer interface, resulting in a thick transition zone as well as aggregated MoS_2_. Moreover, because PDDA is a long chain polymer, it is likely that MoS_2_ and PDDA are loosely packed in the transition zone. On the contrary, during the synthesis of MoS_2_–PrGO where the PDDA was restrained on the PrGO nanosheets, a more ordered assembly between PrGO and MoS_2_ can be formed. Therefore, the membrane resembles a layer-stacked membrane more, with a thinner (and likely denser) transition zone in the bilayer interface.

The thickness of each deposited bilayer was measured by ellipsometry. The density of the LbL membrane was then calculated by using eq 2 with the previously obtained mass measurement. As shown in Figure 4B, regardless of the polycation used, the membrane thickness increased by around 5 nm after depositing each bilayer. Although the thickness measurement shows no obvious differences, a difference in density was observed. As shown in Figure 4C, the density of MoS_2_–PrGO was high during the first three bilayers, then gradually reduced to around 2.2 g/cm^3^. On the other hand, the opposite trend was observed in MoS_2_–PDDA membrane where the density was lower in earlier stage of LbL assembly (around 1.2 g/cm^3^), then increased to around 2.3 g/cm^3^.

The different trends of density change could be attributed to the stacking structure within the LbL membranes. In MoS_2_–PrGO membranes, because the structure resembles layer-stacked membranes, the density value in the first few bilayers is close to the averaged density between dry state MoS_2_ (3.5 g/cm^3^) and PrGO (2 g/cm^3^), which was calculated to be around 2.8 g/cm^3^. Reduced density values observed in the later stages may be attributed to a less regular packing structure, where more defects are generated with more deposited layers.^36^ This measured density is also close to the averaged density between hydrated MoS_2_ and PrGO (2.9 and 1.9 g/cm^3^, respectively, measured by QCM-D), which was calculated to be around 2.4 g/cm^3^. As for the MoS_2_–PDDA membrane, the low density in earlier stages of LbL deposition was likely caused by the loose structure of the LbL layers in the mixed MoS_2_–PDDA region. The increased density in the later stage could be attributed to the increased proportion of MoS_2_ (92% in mass, as shown in Figure 4A), making the membrane density approach that of the hydrated MoS_2_. Note that the density is still less than hydrated MoS_2_, but closer to averaged value of MoS_2_ and PDDA (around 2 g/cm^3^). This observation explains why the thickness change we observed in Figure 4B was similar between MoS_2_–PDDA and MoS_2_–PrGO. During the layer deposition, the MoS_2_ was intertwined with PDDA polymer chain, resulting in a mixed MoS_2_–PDDA region that reduced the overall bilayer thickness as well as the density of MoS_2_ layer.

Compared to MoS_2_–PDDA, the MoS_2_–PrGO membrane showed a much better structural integrity upon drying. From Figure 4C, the dried MoS_2_–PrGO membrane has a density of around 2.3 g/cm^3^ which only increased slightly compared to the hydrated state (2.27 g/cm^3^) indicating that the absence of water molecules between layers does not affect the layer structure significantly. Our calculation suggested that as a result of the density change, the interlayer spacing between stacked PrGO and MoS_2_ only decreased from 1.33 to 1.21 nm upon drying (see Text S6 for detailed calculations). On the other hand, the density of the dried MoS_2_–PDDA film increased from 2 to 3 g/cm^3^, and the interlayer spacing dropped from 1.76 to 0.88 nm correspondingly. This significant change on density and the interlayer spacing informed us that the hydrated MoS_2_–PDDA was indeed relatively loose; hence, after removing the water molecules, the layers will collapse and become denser, leading to restacking.

In summary, the QCM-D and ellipsometry measurements indicated that the MoS_2_–PrGO assembly is more ordered (i.e., the nanosheets are well-aligned), and its structure resembles layer-stacked membranes. On the other hand, because MoS_2_ dominates the MoS_2_–PDDA membrane, regions of loosely packed MoS_2_–PDDA and regions of MoS_2_ aggregates may coexist, potentially making the membrane less stable in water. The XRD analysis in Figure 3A also supports our hypothesis on the structure of those two membranes. In MoS_2_–PrGO, only one peak that corresponds to PrGO-intercalated MoS_2_ was observed, suggesting its resemblance to a layer-stacked membrane. On the other hand, in MoS_2_–PDDA, peaks from both MoS_2_ aggregate and PDDA-intercalated MoS2 were observed.

### 3.5 Swelling Behavior of the MoS_2_–PrGO and MoS_2_–PDDA Membranes

Polyelectrolyte multilayers are often subject to swelling under high ionic strength due to the weakened electrostatic interaction between polycations and anions.^12,37^ The swelling behavior of the MoS_2_–PrGO and MoS_2_–PDDA membranes was monitored by ellipsometry in solutions of different ionic strength. As plotted in Figure 4D, the MoS_2_–PDDA membrane exhibited much more severe swelling than MoS_2_–PrGO in Na_2_SO_4_, reaching around 180% at an ionic strength of 750 mM. The difference in swelling behavior observed between those two membranes is consistent with our previous discussion on the layer structure using XPS and density measurements in Sections 3.3 and 3.4. The swelling of MoS_2_–PDDA was likely attributed to the mixed MoS_2_–PDDA regions formed by the entanglement of MoS_2_ nanosheets with free PDDA at the bilayer interface, where the structure is thick and loose. In comparison, in MoS_2_–PrGO, the mixing between MoS_2_ and PrGO is dense and thin at the interface; thus, it has better resistance to swelling under high ionic strength (120% at ionic strength of 750 mM).

The impact of different ions on swelling behavior was also assessed in 750 mM NaCl and MgCl_2_ solutions, and the MoS_2_–PrGO membrane has better resistance to swelling regardless of the salt species tested (Figure S10A,B). The swelling behavior of the MoS_2_–PrGO membrane in Na_2_SO_4_ and MgCl_2_ was similar over different ionic strength solutions (Figure S10C, the percent swelling increase was similar to increased ionic strength). Note that less swelling was observed in NaCl solutions than in divalent ion solutions, which was possibly due to the lower charge density from the monovalent ions. pH has a negligible effect on membrane swelling under similar ionic strength conditions (Figure S10D), likely attributed to the consistent charge from PrGO and MoS_2_ across a wide pH range (Figure 1C).

### 3.6 Membrane Performance of the MoS_2_–PrGO and MoS_2_–PDDA Membranes

**Figure 5:**
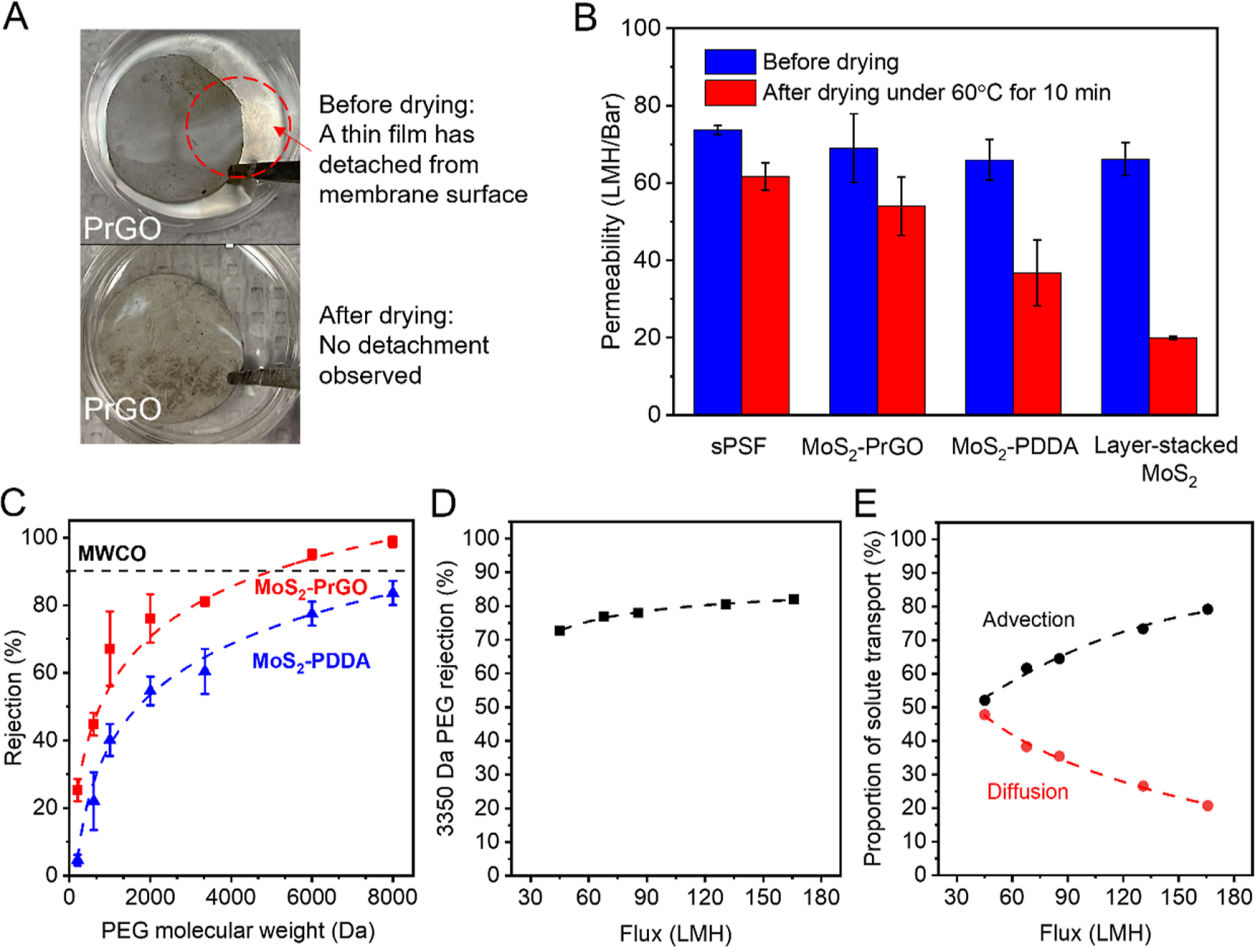
Filtration performance of MoS_2_–PrGO and MoS_2_–PDDA membranes with 9 bilayers. (A) The improvement of membrane stability after drying under 60 °C for 10 min. Before drying, when the LbL membrane was submerged in water, a brown colored thin film was detached from the sPSF substrate (photo on top, circled in red). Photos of MoS_2_–PrGO were shown here as an example, same phenomenon was observed for both MoS_2_–PrGO and MoS_2_–PDDA membranes. (B) The permeability of LbL membranes compared to membrane substrate and layer stacked MoS_2_ membranes. The layer stacked membranes were prepared with the same thickness (around 50 nm) as that of LbL membranes. (C) MWCO for the LbL membranes. The dashed lines were obtained by fitting the data with a logistic model mainly to serve as guidance for visual interpretation. (D) Rejection of 3350 Da PEG as a function of water flux. (E) The proportion of solute transport through advection and diffusion.

Performing a simple drying step is critical to improve the membrane stability. As illustrated in Figure 5A, without any drying step, part of the LbL membrane was detached from the sPSF substrate, and a thin film was observed on the water surface. A video clip documenting the detached LbL membrane is available in the Supporting Information (Video clip #1). This phenomenon was observed in both the MoS_2_–PrGO and the MoS_2_–PDDA membranes. After drying under 60 °C for 10 min the membranes were more stable in water, and no visible detachment was observed. A video clip documenting the stable LbL membrane after drying is available in Supporting Information (Video clip #2). Note that membranes were not completely dried because the pores in sPSF substrate will collapse and lose its permeability when completely dehydrated. The interlayer spacing of the membranes should be identical with completely dried membranes (Figure S11). Future optimization can be done to improve the sPSF stability; however, in the current study we will only focus on the performance of the MoS_2_–PrGO and MoS_2_–PDDA membranes.

The water permeability of the MoS_2_–PrGO membrane was higher than that of MoS_2_–PDDA, especially after performing the drying step. We performed preliminary tests on membranes composed of 6, 9, and 12 bilayers and found that they follow the permeability-selectivity trade-off trend (Figure S12), i.e., increasing number of bilayers resulted in less water permeability but better selectivity. We selected the 9-bilayer membrane as a representative in remaining filtration tests. As shown in Figure 5B, the 9-bilayer MoS_2_–PrGO and MoS_2_–PDDA membranes before drying had very similar permeability as the sPSF substrate, which was around 70 LMH/Bar. However, after the drying step, the MoS_2_–PrGO membrane demonstrated higher permeability (around 55 LMH/bar) than the MoS_2_–PDDA membrane (around 40 LMH/bar). The loss of permeability of the MoS_2_–PrGO membrane was likely due to the densification of sPSF substrate upon drying, as both have lost around 15 LMH/bar after the drying step. The additional permeability loss in MoS_2_–PDDA membranes is likely due to the restacking of the aggregated MoS_2_, which is known to occur in layer-stacked MoS_2_ nanosheets.^2^ The higher permeability observed from the MoS_2_–PrGO membrane indicates that compared to PDDA, PrGO is more effective in intercalating between MoS_2_ nanosheets and preventing MoS_2_ from restacking. Additionally, the surface contact angle measurement for both membranes indicates that the membranes have similar hydrophilicity (Figure S13), which is thus unlikely the key factor resulting in the difference in membrane permeability. It is worth noting that after drying, both LbL membranes had better permeability than pure layer stacked MoS_2_ membranes (around 25 LMH/bar) prepared with the same membrane thickness. This indicates that regardless of the polycation used, LbL is an effective strategy to reduce the restacking of MoS_2_.

The separation capability of the membranes was tested by evaluating their MWCO using PEGs with various molecular weight. As presented in Figure 5C, the MoS_2_–PrGO has a MWCO at around 5000 Da, which is lower than the MoS_2_–PDDA membrane (more than 8000 Da.). The higher MWCO of MoS_2_–PDDA membranes could be attributed to the MoS_2_ aggregates in the MoS_2_–PDDA membranes, which can introduce structural defects within the LbL layer and have negative effects on membrane selectivity.^30^ For both membranes, the rejection for PEG with a higher molecular weight increased gradually. This indicates that the LbL membrane has a wide distribution of pore sizes, possibly due to the imperfect alignment between nanosheets.

The transport of the solute within the LbL membranes is likely dominated by advection. To understand the proportion of advective and diffusive solute transport, we examined the rejection of 3350 Da of PEG at different water fluxes. The results for 9-bilayer MoS_2_–PrGO membranes were shown here as an example. As shown in Figure 5D, the rejection increased from around 70% at 40 LMH to 80% at 80 LMH. When the flux was further increased, the rejection remained almost constant, indicating a dominant advective transport or pore flow in the MoS_2_–PrGO membrane. By modeling the data, we can determine the advection coefficient (α) and diffusion coefficient (*B*) and calculate the solute flux from advection and diffusion (see Text S7 and Figure S14 for detailed calculations). The proportion of solute flux from each mechanism is shown in Figure 5E. As seen in the plot, the advection accounted for around 50% of the total solute flux at lower water flux, and it increased to almost 80% when the flux increased, indicating that advection is the dominant transport mechanism of the solute.

**Figure 6:**
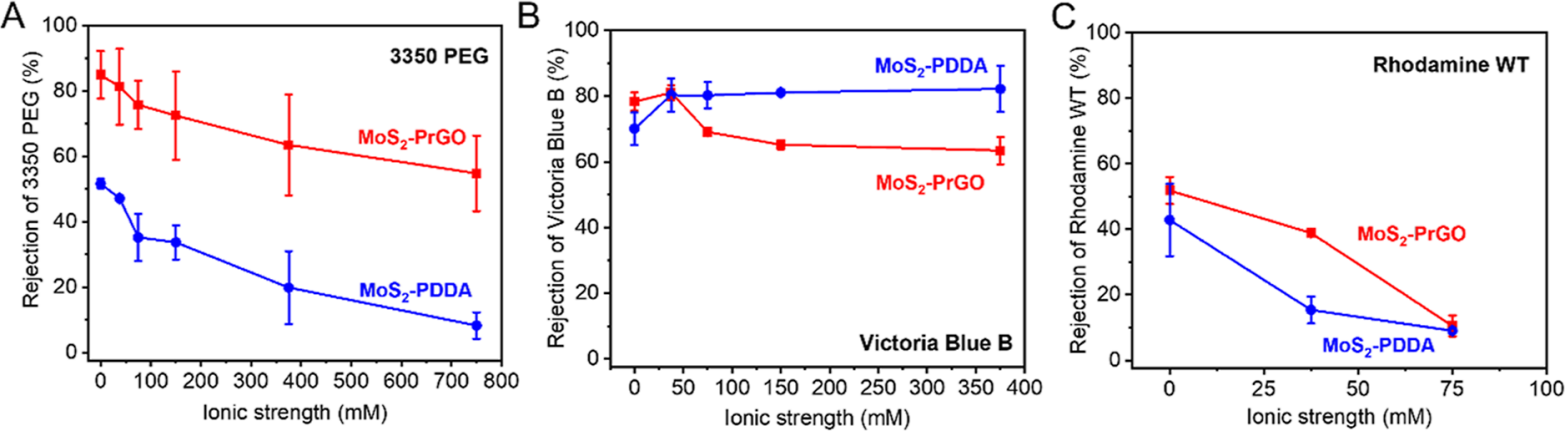
Rejection of 3350 Da PEG (A), VB (B), and RWT (C) by the MoS_2_–PrGO and MoS_2_–PDDA membranes at different ionic strength. The ionic strength was adjusted to 7.5, 15, 37.5, 75, 150, 375, and 750 mM by using sodium chloride. Membranes with 9 bilayer deposition were used for all tests.

To examine the effect of membrane swelling on selectivity, we also performed rejection tests for PEG with a molecular weight of 3350 Da under a higher ionic strength. As shown in Figure 6A, the PEG rejection by MoS_2_–PrGO membrane decreased by 25% when ionic strength increased to 750 mM, while for the MoS_2_–PDDA membranes, there was a 43% decrease in rejection. This correlates well with our previous swelling tests, which demonstrated more severe swelling in the MoS_2_–PDDA membrane (about 180%) than in the MoS_2_–PrGO membrane (about 120%) at an ionic strength of 750 mM.

To understand the charge effects on the separation performance of the membranes, we performed rejection tests using a positively charged dye, VB, and a negatively charged dye, RWT, under different ionic strength conditions. As shown in Figure 6B, at ionic strength below 7.5 mM, the rejection of the VB by MoS_2_–PrGO was around 80%, which was better than the MoS_2_–PDDA membranes (70%), possibly due to the better size exclusion effect from the well-aligned MoS_2_–PrGO. However, with an increase in ionic strength, the VB rejection by the MoS_2_–PDDA membrane improved slightly to 80%, while the VB rejection by the MoS_2_–PrGO membrane gradually decreased to 60%. This indicates that the overall charge of MoS_2_–PrGO was more negative than MoS_2_–PDDA, leading to weaker charge repulsion effects toward the positively charged VB than MoS_2_–PDDA, hence exhibiting worse rejection at higher ionic strength due to the charge screening effect. As for the negatively charged RWT, the rejection rate of MoS_2_–PrGO was better than MoS_2_–PDDA at lower ionic strengths which can also be attributed to its stronger negative charge (Figure 6C). However, both membranes exhibited lower rejection to RWT compared to VB. Because the chemical structure and the molecular weight of those two dyes are similar (Figure S15), the difference observed for rejection should mainly be attributed to charge effects, but not size exclusion. Because the zeta-potential of PDDA is higher than MoS_2_,^38^ it is possible that PDDA provided higher charge density than MoS_2_ within the LbL structure, resulting in better rejection to VB than negatively charged RWT. Similar phenomenon was also observed in previous studies. Liu et al. and Yan et al. found that, graphene oxide-polyelectrolyte LbL membrane with negatively charged surface had better rejection toward positively charged ions and dye molecules.^14,39^

Lastly, we also examined the long-term performance of the MoS_2_–PrGO membranes. The membranes were continuously tested for 24 h after being stored for 1–2 weeks. As shown in Figure S16, no significant change of rejection was observed after 24 h of operation and 1–2 weeks storage, suggesting the MoS_2_–PrGO membranes remained stable. Although the water flux during 24 h of test slightly declined, it is most likely due to the pore-blocking effects from PEG molecules during filtration.

We have shown that the LbL membranes made by using nanosheets as both polycation and polyanion have better stability than those made by using nanosheet-polymer pairs. The LbL assembly also improved the selectivity of UF membranes without sacrificing membrane permeability. We compared the performance of our membrane with data from previous studies on nanosheet-based LbL membranes in Figure S17 and Table S1.^2,41−50^ Note that most studies focused on nanosheet-polymer LbL membranes for RO/NF and their antifouling and chlorine resistance properties.^8,15,50^ While some studies also investigated their ability to improve membrane selectivity, it is often associated with sacrificed permeability.^14,46,47^ Our membrane is one of the few works that uses LbL assembly to improve the performance of UF membranes.

As an outlook for future work, we think that the LbL assembly of 2D nanosheets has a great potential to help improve the performance of UF membranes. However, the LbL films tend to have a loose structure and a wide distribution of pore sizes, resulting in compromised membrane selectivity. This loose structure may be attributed to the uneven surface roughness and charge density of the membrane substrate and the unrefined procedure of LbL deposition. Improving substrate quality and carefully optimizing the synthesis procedure (e.g., transferring the membrane between material solutions without disturbing the deposited LbL films) may help improve membrane selectivity. Another challenge in synthesizing LbL membranes is the limitation of the maximum number of bilayers that can be deposited. Having a thicker layer is usually beneficial for improving selectivity. However, we found that after around 12 bilayers, the membranes start to delaminate, resulting in compromised filtration performance. It thus would be beneficial to add stabilization steps (e.g., drying the membranes for every 6 bilayers of deposition) during LbL if a thicker membrane is desired.

## 4 Conclusions

In this study, we have investigated the structure and properties of two types of LbL-assembled MoS_2_ membranes prepared with either nanosheets or a polymer-based polyelectrolyte as polycation. PDDA-functionalized rGO (PrGO) was prepared as a nanosheet-based polycation to be assembled with MoS_2_ to make MoS_2_–PrGO membranes. Meanwhile, PDDA was also directly used as a polymer-based polycation to create MoS_2_–PDDA membranes. PrGO was proved to be more effective than PDDA in controlling the restacking of MoS_2_ when dried, because the nanosheet-based polycation (PrGO) creates a much better aligned structure with MoS_2_ nanosheets in the LbL assembly due to their similarity in shape, size, and charge density. Accordingly, the MoS_2_–PrGO membrane showed better stability under high ionic strength and was able to deliver higher water flux with a better size exclusion ability. On the other hand, the MoS_2_–PDDA membrane contained regions of loosely packed MoS_2_–PDDA aggregates that are susceptible to swelling. At the same time, regions of MoS_2_ aggregates also exist, making the membrane susceptible to restacking when being dried. Overall, we have shown that the membranes made by LbL assembly of 2D nanosheets with matching properties deliver better structural stability and filtration performance than using polymer-based polycations, bringing the MoS_2_-based membrane one step closer to being used in real filtration systems.

## Supporting Information Available

XPS, XRD, and swelling tests; filtration tests and membrane characterizations; literature review; experimental procedures for graphene oxide preparation, XRD sample preparation, combined QCM-D and ellipsometry characterization, and Dragendorff method for colorimetric characterization of PEG concentration; supporting calculations for material loading and interlayer spacing; and supporting calculations for solute transport (PDF)
Video clip #1 documenting layer detachment from the LbL membrane before drying (MOV)
Video clip #2 documenting stable LbL membrane after drying (MOV)

## Supplementary Material

am4c03891_si_001.pdf

am4c03891_si_002.mov

am4c03891_si_003.mov
