# Supplementary material for: Layer-by-layer Assembly of Nanosheets with Matching Size and Shape for More Stable Membrane Structure than Nanosheet-Polymer Assembly
Source: ACS Appl Mater Interfaces. 2024 May 8;16(20):26568–79. doi: 10.1021/acsami.4c03891 (PMC11129114; doi:10.1021/acsami.4c03891)
Supplement: Supplementary file 1 — am4c03891_si_001.pdf [file am4c03891_si_001.pdf]

Supporting Information

# Layer-by-Layer Assembly of Nanosheets with Matching Size and Shape for More Stable Membrane Structure Than Nanosheet-Polymer Assembly

*ACS Applied Materials & Interfaces*

Monong Wang[a], Young-Jin Song[a], Wenli Jiang[a], Francesco Fornasiero[b], Jeffrey J. Urban[c], Baoxia Mi[a*]

[a] Department of Civil and Environmental Engineering, University of California, Berkeley, California 94720, United States

[b] Biosciences and Biotechnology Division, Lawrence Livermore National Laboratory, Livermore, CA 94550, United States

[c] Molecular Foundry, Lawrence Berkeley National Laboratory, Berkeley, CA 94720, United States

[*] The author to whom correspondence should be addressed. E-mail: mib@berkeley.edu; Tel.: (510) 664-7446.

**Supporting Figures** ........................................................................................................ 3
Figure S1. XPS results for LbL materials. ..................................................................... 3
Figure S2. Physical properties of PrGO. ....................................................................... 4
Figure S3. XPS Mo 3d scan for chemically exfoliated $MoS_2$. ......................................... 4
Figure S4. Thickness and size of $MoS_2$. ....................................................................... 5
Figure S5. Image of $MoS_2$-PDDA and $MoS_2$-PrGO LbL membranes with different numbers of layer deposited. ............................................................................................................... 5
Figure S6. S 2p scan for $MoS_2$-PDDA membranes with different number of bilayers. ............ 5
Figure S7. The Mo3d scan for $MoS_2$-PrGO and $MoS_2$-PDDA membranes with different number of bilayers. ............................................................................................................. 6
Figure S8. Mass and thickness of $MoS_2$-PDDA and $MoS_2$-PrGO films during layer deposition. ............................................................................................................................. 6
Figure S9. PDDA weight percent of $MoS_2$-PrGO and $MoS_2$-PDDA films. ............................. 7
Figure S10. Swelling of $MoS_2$-PrGO and $MoS_2$-PDDA. ........................................................ 7
Figure S11. XRD of pure PrGO and $MoS_2$ before and after drying for 10 min under 60°C. .... 8
Figure S12.Filtration perforamnce for 6, 9, and 12 bilayers of $MoS_2$-PDDA and $MoS_2$-PrGO membranes before drying. ..................................................................................................... 8
Figure S13. Surface contact angle of $MoS_2$-PrGO and $MoS_2$-PDDA with 6 bilayers. ............ 8
Figure S14. Determination of the advection coefficient by correlating rejection and water flux. ............................................................................................................................................ 9
Figure S15. Structure of Victoria Blue B (VB) and Rhodamine WT (RWT) drew by MolView. ............................................................................................................................ 9
Figure S16. Long-term filtration performance of $MoS_2$-PrGO membrane. ............................ 10
Figure S17. Filtration performance of nanosheet-based membranes from previous studies. .. 10
**Supporting Table**
Table S1. Filtration performance of nanosheet-based membranes from previous studies. ..... 10
**Supporting Experimental Procedure** ......................................................................... 12
Text S1. Preparation of GO nanosheets ......................................................................... 12
Text S2. Preparation of XRD samples ............................................................................ 12
Text S3. Characterizing mass loading during layer-by-layer synthesis using QCM-D and ellipsometry ......................................................................................................................... 12
Text S4. Determination of PEG concentration using Dragendorff method ............................. 13
**Supporting Calculations** .............................................................................................. 14
Text S5. Calculating the loading of PDDA on PrGO ............................................................ 14
Text S6. Calculating the interlayer spacing of LbL membranes ............................................. 14

## Supporting Figures

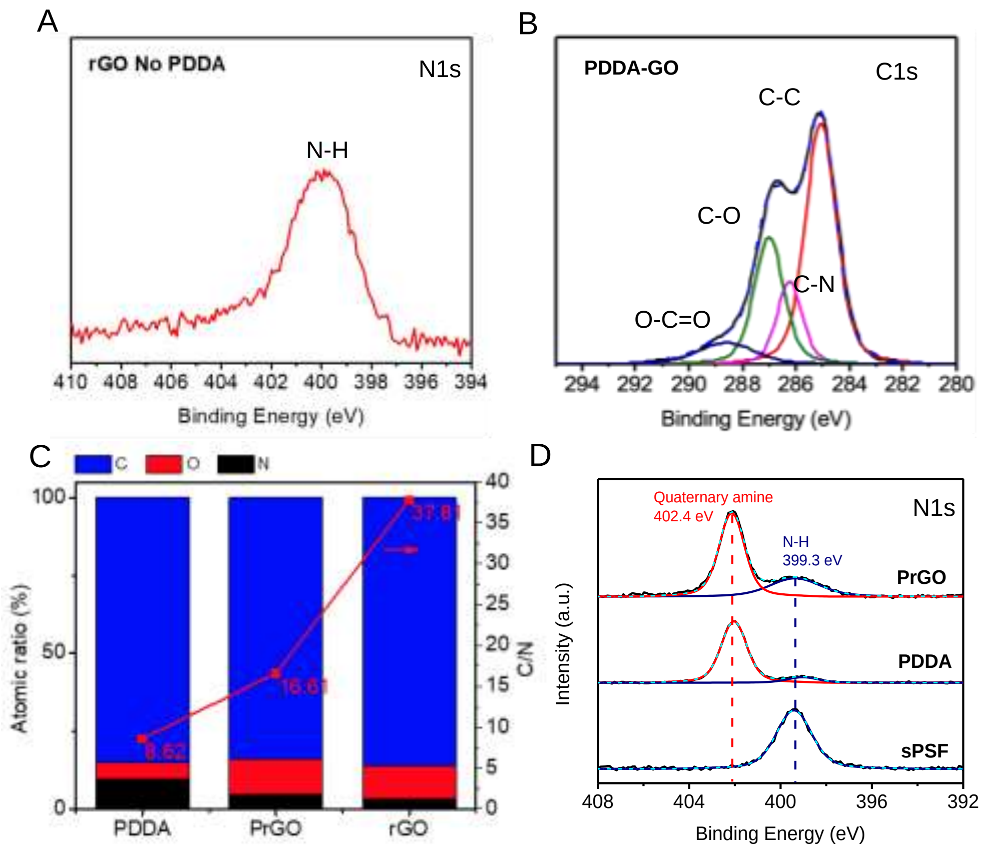


Figure S1. XPS results for LbL materials. (A) N 1s scan of rGO. The rGO without PDDA functionalization only has one peak that belongs to the N-H bond (399.3 eV). (B) C 1s scan of PDDA-GO. GO nanosheets functionalized with PDDA without reduction have higher intensity at the C-O peak. (C) Atomic ratio of C, O and N in PDDA, PrGO and rGO. (D) N 1s scan for PDDA, sPSF substrate and PrGO. Only PDDA and PrGO carries the peak from quaternary amine (402.4 eV).

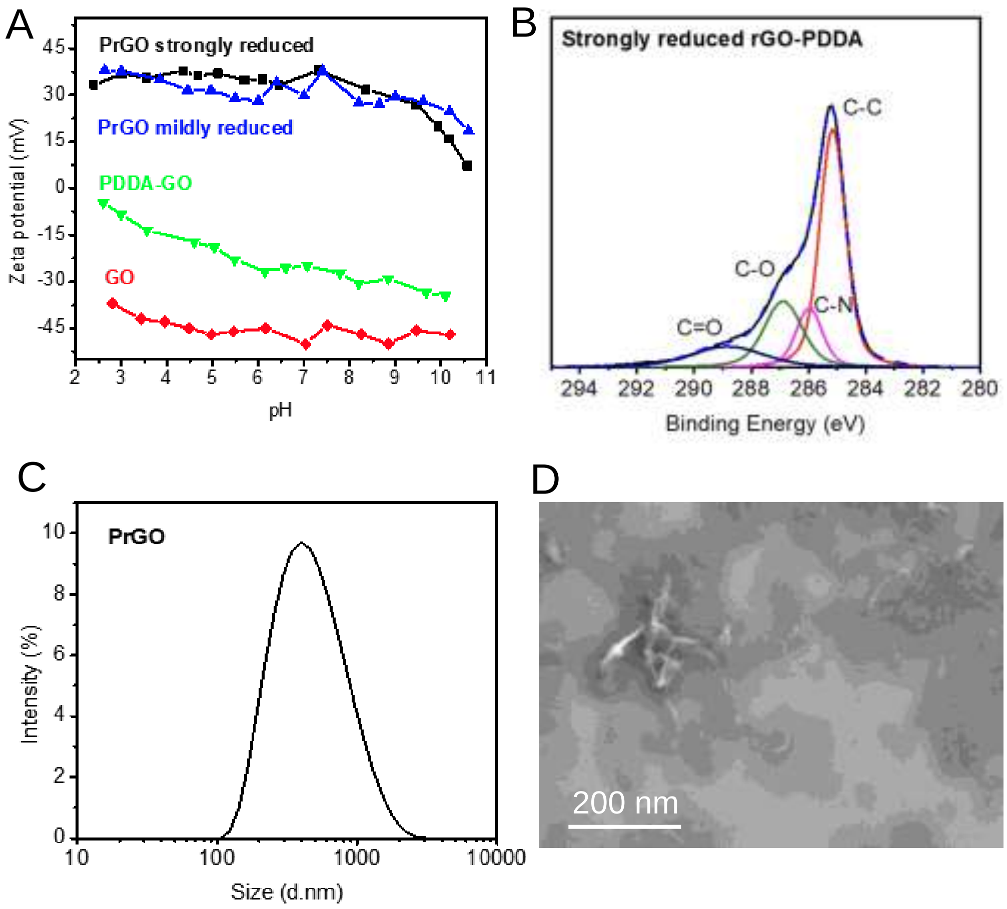


Figure S2. Physical properties of PrGO. (A) Charge comparison between strongly reduced (black line) and mildly reduced GO (blue line). 2 times higher concentration of hydrazine and ammonia were used to synthesize the strongly reduced PrGO. No significant difference on zeta potential was observed between those two types of PrGO. (B) C 1s scan of strongly reduced rGO-PDDA. (C) Size of PrGO suspension measured using Zetasizer. (D) SEM image for drop cast PrGO.

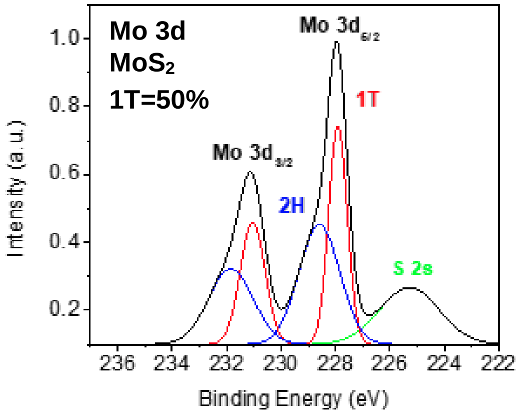


Figure S3. XPS Mo 3d scan for chemically exfoliated $MoS_2$.

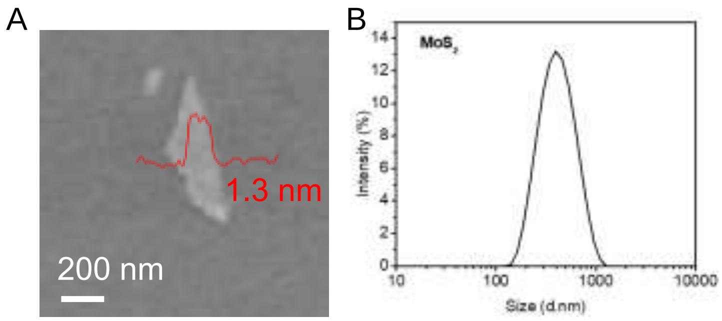


Figure S4. (A) Thickness of chemically exfoliated $MoS_2$ measured by AFM. (B) Size of $MoS_2$ measured by Zetasizer.

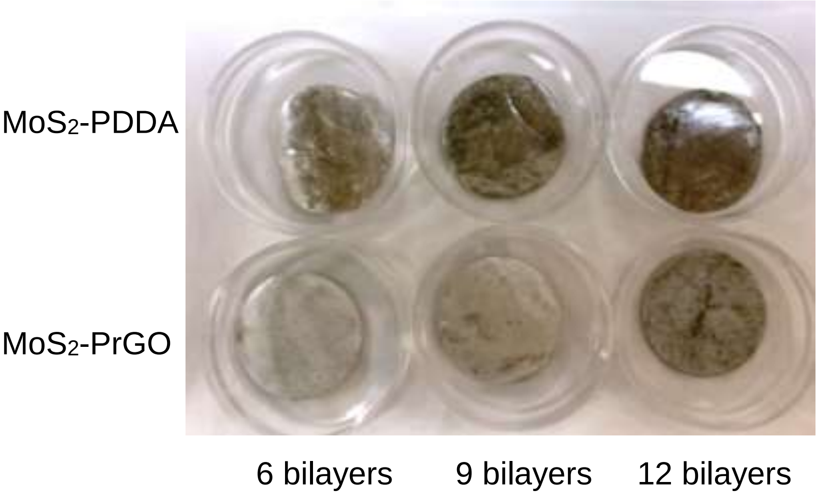


Figure S5. Image of $MoS_2$-PDDA and $MoS_2$-PrGO LbL membranes with different numbers of layer deposited.

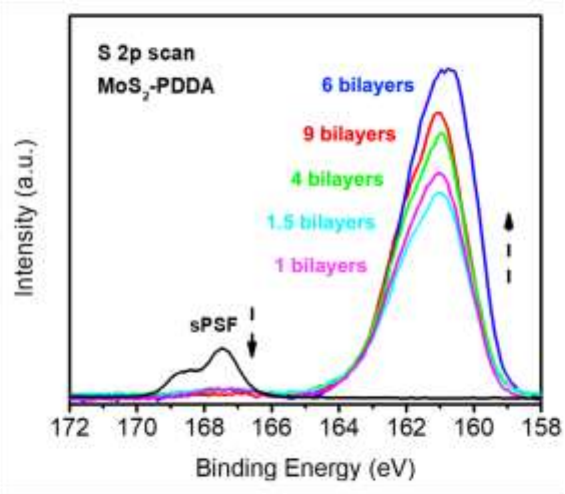


Figure S6. S 2p scan for $MoS_2$-PDDA membranes with different number of bilayers. The S 2p peak from sPSF at 167.5 eV disappeared after 4 deposited bilayers indicating complete coverage of the sPSF by the bilayers. The S 2p peak from $MoS_2$ at 161 eV was intensified with more bilayers at the same time.

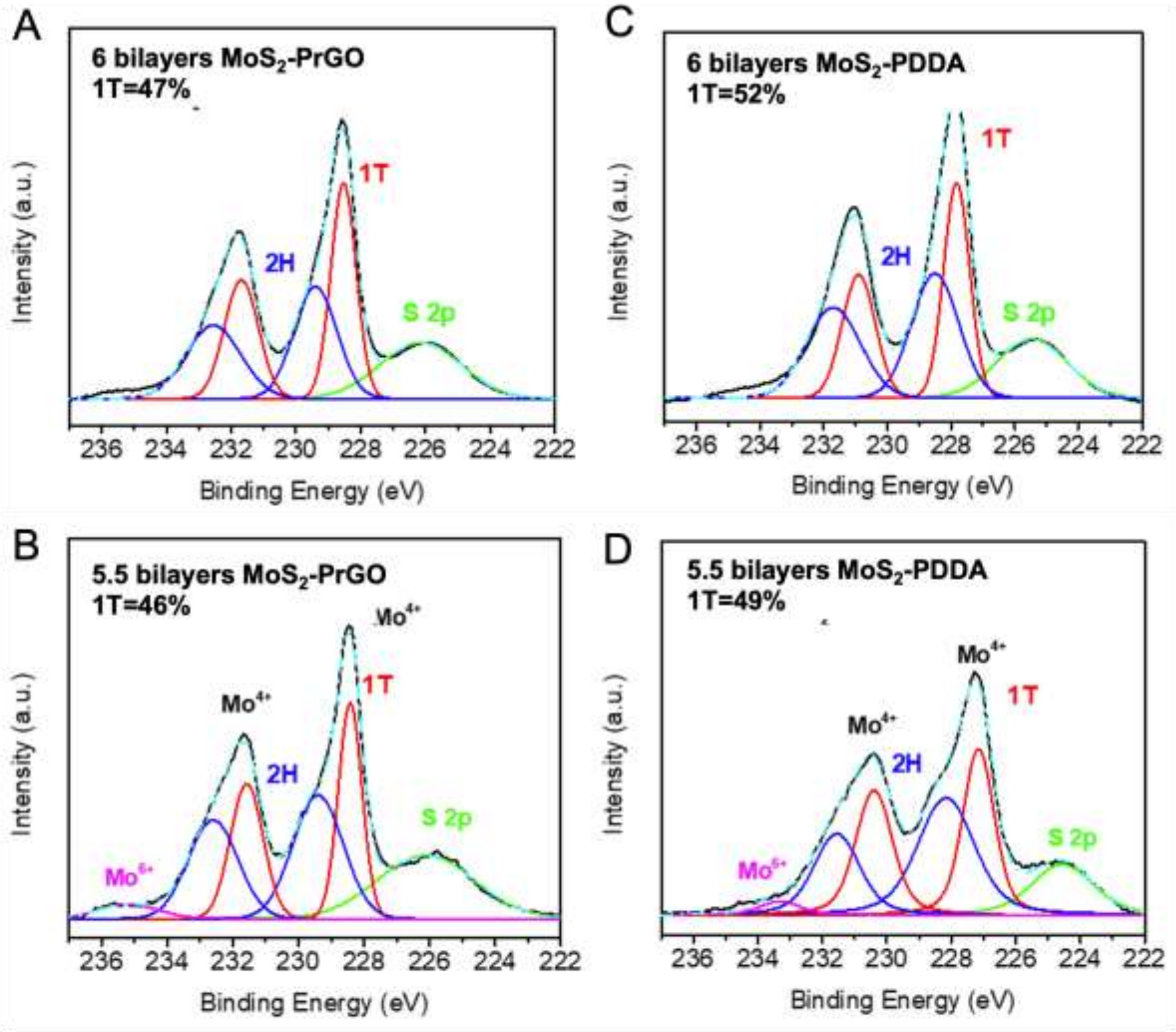


Figure S7. The Mo3d scan for $MoS_2$-PrGO with (A) 6 and (B) 5.5 bilayers, and $MoS_2$-PDDA membranes with (C) 6 and (D) 5.5 bilayers.

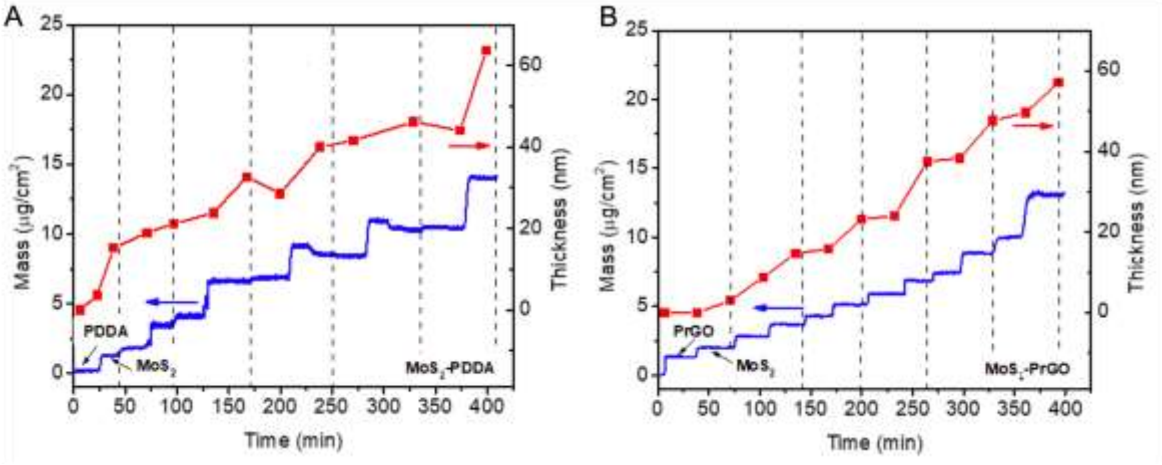


Figure S8. (A) Mass (measured by QCMD) and (B) thickness (measured by ellipsometry) of $MoS_2$-PDDA and $MoS_2$-PrGO films during layer deposition.

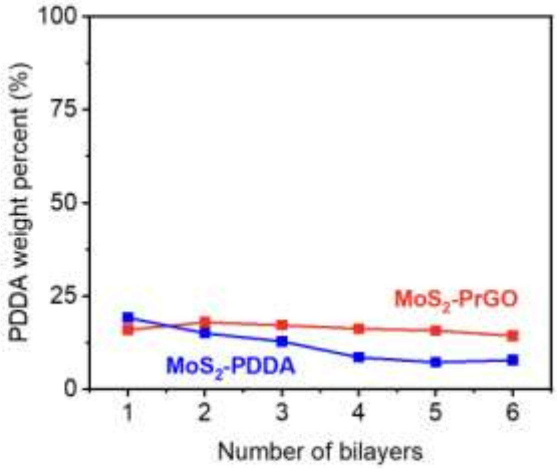


Figure S9. PDDA weight percent of $MoS_2$-PrGO and $MoS_2$-PDDA films.

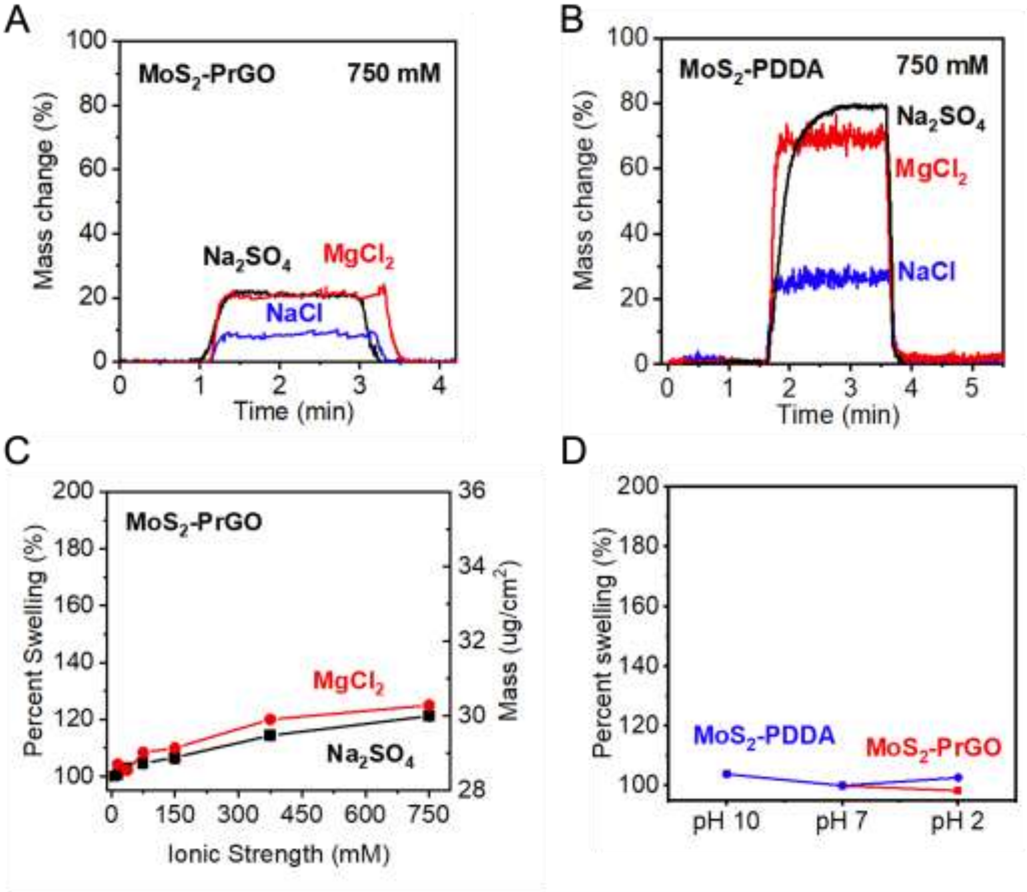


Figure S10. Swelling of (A) $MoS_2$-PrGO and (B) $MoS_2$-PDDA films in 750 mM NaCl, $Na_2SO_4$ and $MgCl_2$ solutions. (C) Swelling of $MoS_2$-PrGO films with $Na_2SO_4$ and $MgCl_2$ at different ionic strength. (D) Swelling of $MoS_2$-PrGO and $MoS_2$-PDDA films in different pH at ionic strength of 10 mM.

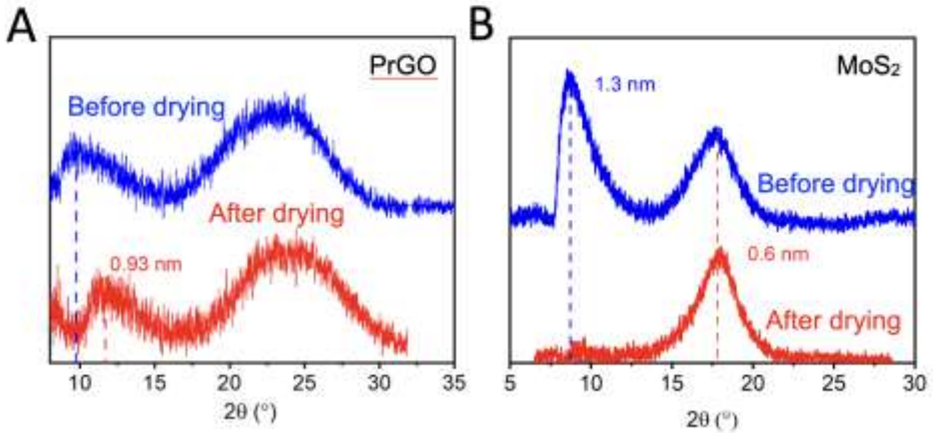


Figure S11. XRD of pure A. PrGO and B. MoS2 before and after drying for 10 min under 60°C. The interlayer spacing of the materials after drying for 10 min is the same as completely dried condition.

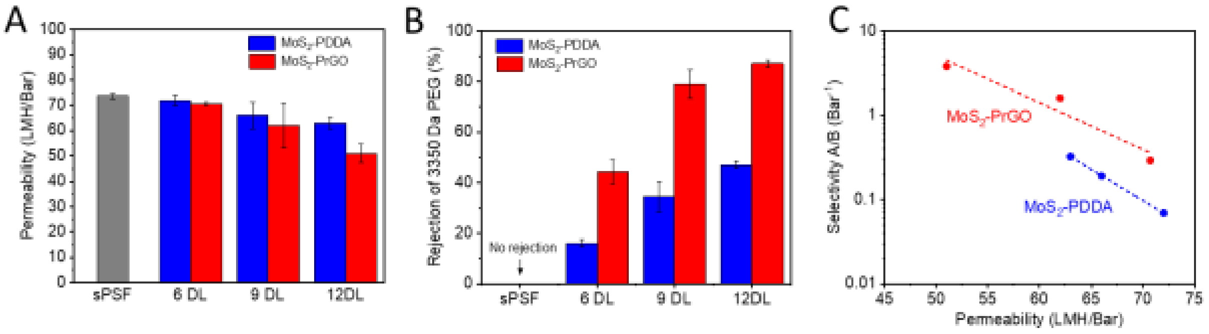


Figure S12. (A) Permeability, (B) rejection of 3350 Da PEG, and (C) selectivity plotted against permeability for 6, 9, and 12 bilayers of $MoS_2$-PDDA and $MoS_2$-PrGO membranes before drying.

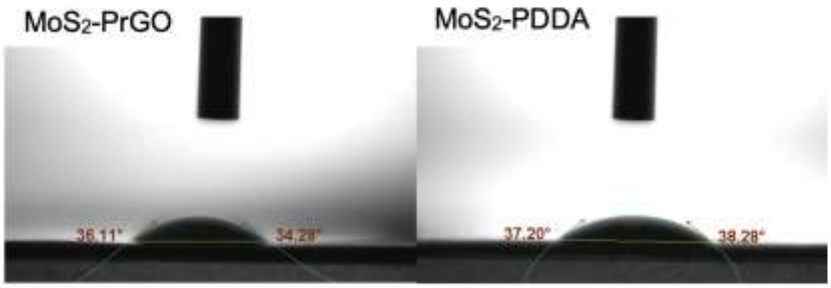


Figure S13. Surface contact angle of $MoS_2$-PrGO and $MoS_2$-PDDA with 6 bilayers.

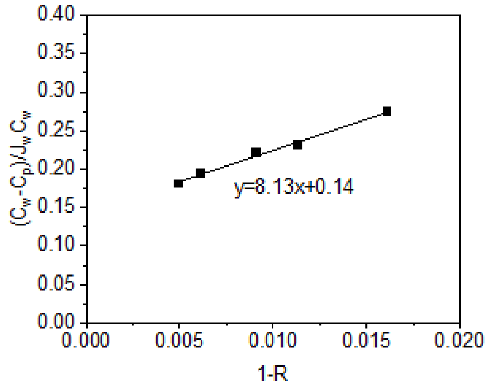


Figure S14. Determination of the advection coefficient by correlating rejection and water flux. The the y-intersect is the advection coefficient,$\alpha$, and the slope is the diffusion coefficient, B.

Victoria Blue B
pka=~2
Mw=506

Rhodamine WT
pka=~4.5
Mw=567

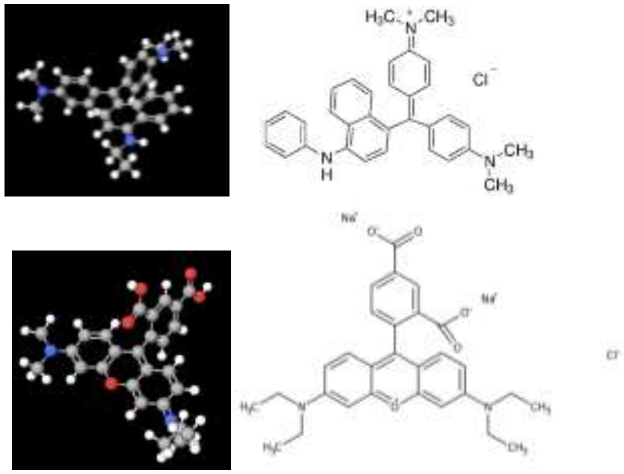

Figure S15. Structure of Victoria Blue B (VB) and Rhodamine WT (RWT) drew by MolView. The chemical composition for VB was obtained from Sigma Aldrich. The chemical composition for RWT was from Fisher scientific.

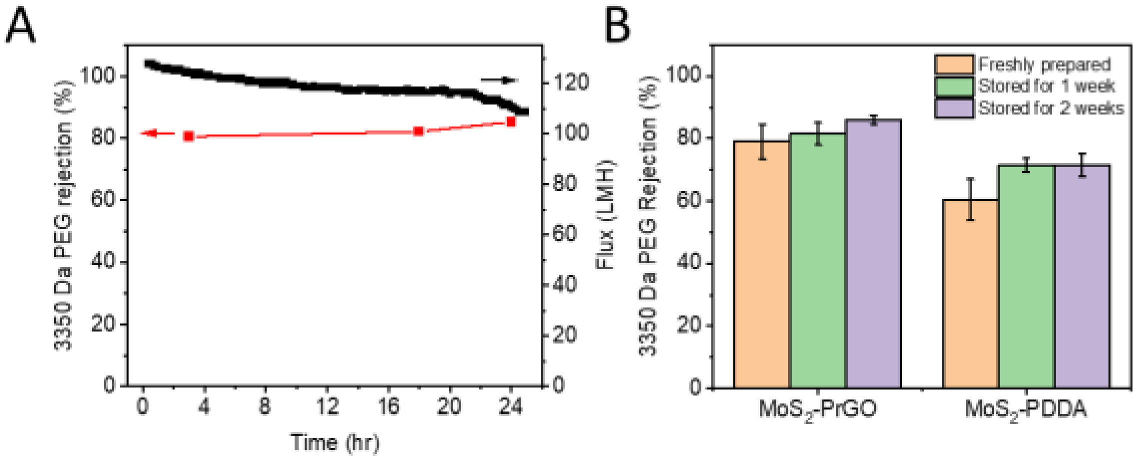


Figure S16. Long-term filtration performance of $MoS_2$-PrGO membrane. A. Flux and rejection after continuous operation for 24 hours. B. Rejection after storing the membranes for 1 and 2 weeks.

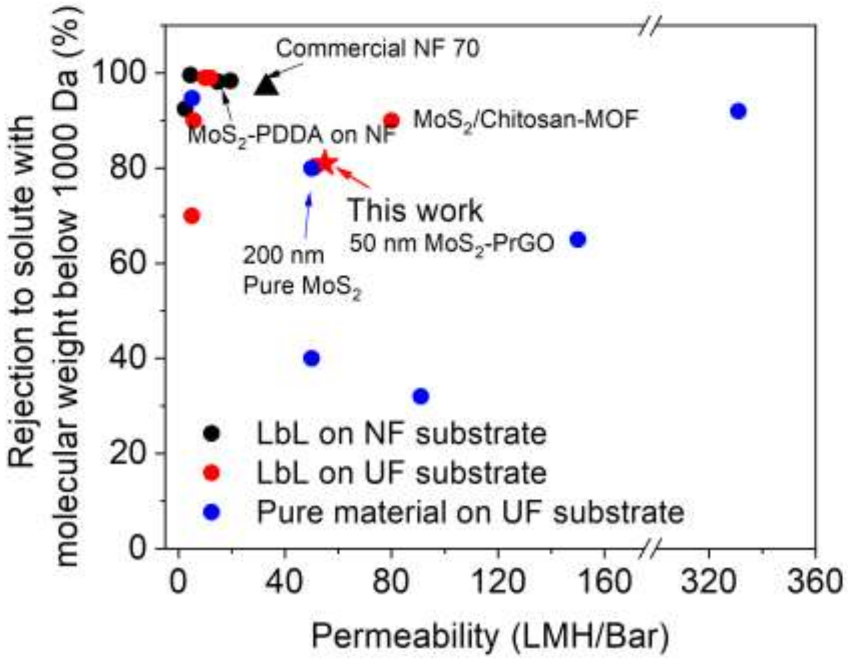


Figure S17. Filtration performance of nanosheet-based membranes from previous studies. The permeability results were selected from membranes with thickness less than 500 nm. The rejection results were selected for feed molecules with a molecular weight less than 1000 Da. Detailed information are summarized in Table S1.

**<u>Supporting Table</u>**

**Table S1**

| **LbL on NF substrate** | | | | |
|---|---|---|---|---|
| **Type of membrane** | **Permeability (LMH/Bar)** | **Rejection (%)** | **Feed molecule** | **References** |
| PAH/Graphene oxide | 2.5 | 92.5 | methelene blue | [1] |
| Graphene oxide /PDDA/PAN | 14.8 | 98.2 | methelene blue | [1] |
| PDDA/Graphene oxide | 4.5 | 99.6 | methelene blue | [1] |
| PDDA/$MoS_2$ | 19.5 | 98.4 | methelene blue | [2] |
| PEI/Graphene oxide | 4 | 75 | $MgCl_2$ | [3] |
| NF 70 (typical range for dye rejection) | 33 | 97 | | [3] |
| **LbL on UF substrate** | | | | |
| $MoS_2$/CMOF | 80 | 90 | 600 Da PEG | [4] |
| PDDA/$WS_2$/PAA | 5.72 | 90 | 1000 Da PEG | [5] |
| Mxnex/glutaric acid | 12 | 99 | methelene blue | [6] |
| $MoS_2$-PrGO | 55 | 81 | Victoria Blue B | This study |
| **Pure Material prepared through vacuum filtration** | | | | |
| $WS_2$ | 331 | 92 | Evans blue | [7] |
| $WS_2$ | 704 | 82 | Evans blue | [7] |
| $MoS_2$ | 32 | 91 | Congo red | [8] |
| $MoS_2$ | 50 | 80 | Rhodamine WT | [9] |
| Mxene | 5.1 | 94.7 | Methelene blue | [10] |
| Mxene-$MoS_2$ composite | 150 | 65 | Methelene blue | [11] |

## Supporting Experimental Procedure

### Text S1. Preparation of GO nanosheets

We prepared GO from graphite using the modified Hummers method[1, 2]. First, graphite flakes were oxidized in a mixture of $KMnO_4$, $H_2SO_4$, and $NaNO_3$ (Sigma-Aldrich, St. Louis, MO). The resulting pasty solution was then diluted and sifted through a polyester nonwoven fabric filter (PET, grade 3249, Ahlstrom, Helsinki, Finland) by vacuum filtration. The GO solids retained by the fabric filter were suspended in deionized (DI) water and centrifuged at 8000g-force using a Sorvall RC 6+ (Thermo Scientific, Marietta, OH). The GO solids remaining at the bottom of the centrifuge tube went through at least three cycles of resuspension in DI water and then centrifugation to completely wash out chemical residuals. The washed GO suspension was subsequently ultrasonicated (S-4000, Misonix, Farmingdale, NY) to exfoliate GO particles into GO nanosheets. As the last step, the sonicated solution was centrifuged at 8000g-force to remove any unexfoliated graphite residues, resulting in a pure GO nanosheet suspension.

### Text S2. Preparation of XRD samples

After LbL assembly, free standing LbL films can be collected by first drying the membrane in air for 1 min, then resubmerging in water (see an example photo below, the brown colored thin film was the detached LbL film). The floating LbL film was then collected on a silica wafer and dried with nitrogen gas. To obtain thicker layers for XRD analysis, the procedure was repeated multiple times.

The XRD samples for layer stacked PrGO and $MoS_2$ was prepared by drop casting the solutions on silica wafer.

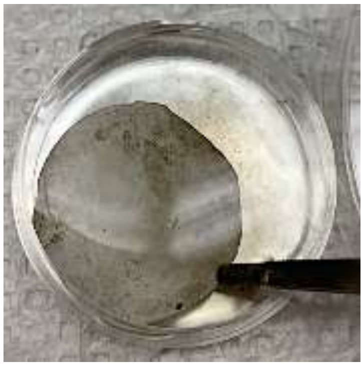

*Image was sharpened for better demonstration

### Text S3. Characterizing mass loading during layer-by-layer synthesis using QCM-D and ellipsometry

The mass deposition of the LbL membrane was monitored and characterized in a multi-chamber QCM-D system (E-4, Q-sense, Sweden). A gold-coated QCM-D sensor was cleaned and treated in a UV/ozone chamber to make its surface negatively charged. First, Milli-Q grade water (MQ) was pumped into the chamber to obtain an equilibrated baseline of the vibration frequency of the clean sensor. Then, 0.5 g/L PrGO or PDDA solution was pumped into the chamber with a flow rate of 0.1 ml/min. The positively charged material was therefore deposited in situ on the QCM-D sensor by electrostatic attraction. To ensure a maximized deposition, the solution was not changed until the frequency of the gold sensor was stabilized. MQ water was subsequently pumped into the chamber at the same flow rate to remove excess polycation. Similar procedure was performed for

$MoS_2$ deposition. The QCM-D measures the change of frequency and dissipation during deposition and the mass of each layer was later calculated using a viscoelastic model.

To analyze the swelling behavior of the as synthesized membrane, the simultaneous QCM-D and ellipsometry measurements were enabled by mounting a single–chamber QCM-D system (E-1, Q-sense, Sweden) onto the sample holder of a multi-wavelength ellipsometer (FS-1Multi-wavelength, Film Sense, Lincoln, NE). The mass change was monitored using QCM-D as described previously. During layer deposition, the ellipsometer measures the change of reflected light polarization from the LbL layers. The measured polarization data was used to fit the refractive index "n" with the Cauchy dispersion model that is built-in in the Film-Sense software (Film Sense, Lincoln, NE), and the thickness of the LbL membranes was calculated.

**Text S4. Determination of PEG concentration using Dragendorff method**

The PEG concentration in membrane feed, retentate and permeate water was determined using Dragendorff method. Dragendorff reagent was prepared by mixing 1) 2.5 mL 16 g/L BiONO3 in 20% acetic acid solution, 2) 2.5 mL 40 g/L potassium iodide solution, and 3) 45 mL 3.88 mol/L acetic acid solution. 0.5 ml of sample containing PEG was mixed with 0.6 mL 8.66 mol/L acetic acid solution and 0.1 mL of the Dragendorff reagent in a standard plastic cuvette. The mixed solution should be transparent with light orange color. The solution was tested after 15 min with UV-Vis spectrophotometer (UV160U, Shimadzu Scientific Instruments, Columbia, MD) at 520 nm. The calibration curve for PEG with different molecular weights can be found in Figure S18.

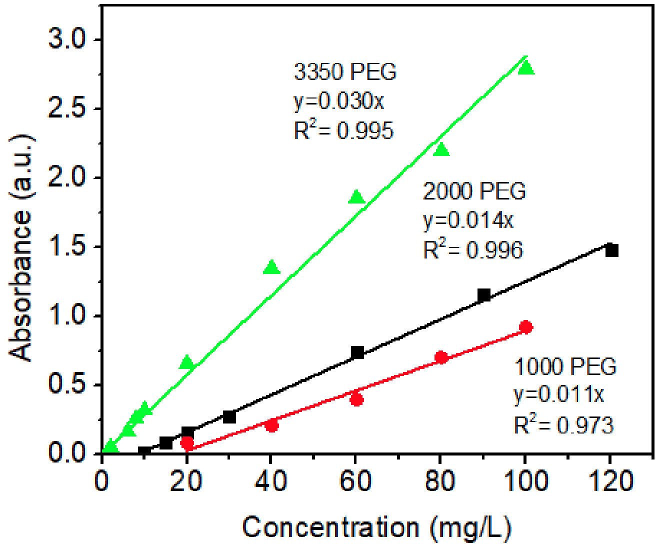


Figure S18. Calibration curves for 3350, 2000, and 1000 MW PEG.

## Supporting Calculations

### Text S5. Calculating the loading of PDDA on PrGO

The chemical composition determined by XPS measurement (Figure S1) was used to estimate the loading of PDDA on PrGO. Using the elemental ratio of C and N measured by XPS, the PDDA can be expressed as $(C_{8.62}O_{0.51}N)_n$ (close to the theoretical value of PDDA monomer $(C_8H_{16}NCl)_n$[12], rGO can be expressed as $(C_{37.81}O_{5.24}N)_n$, and PrGO can be expressed as $(C_{16.61}O_{2.69}N)_n$. By using the C/N ratio of each substance, the number of PDDA chains attached to rGO, $a$, can then be calculated by:

$$\frac{a \cdot N_{PDDA} + N_{rGO}}{a \cdot C_{PDDA} + C_{rGO}} = \frac{N_{PrGO}}{C_{PrGO}}$$

where $N_x$ is the number of nitrogen atoms of substance x (either PDDA, rGO or PrGO), $C_x$ is the number of carbon atoms of substance x. By plugging in the chemical expression for PDDA, PrGO and rGO, the n can be calculated as

$$\frac{a+1}{a \cdot 8.62 + 37.81} = \frac{1}{16.61} \rightarrow a = 2.56 \frac{mol\ PDDA}{mol\ rGO}$$

To calculate the mass ratio:

$$\frac{M_{PDDA}}{M_{PrGO}} = \frac{M_{PDDA}}{M_{rGO} + M_{PDDA}} = \frac{2.56 * 125.66}{690 + 2.56 \cdot 125.66} = 0.32$$

### Text S6. Estimating the interlayer spacing of LbL membranes

The interlayer spacing between the stacked nanosheets in $MoS_2$-PrGO and $MoS_2$-PDDA membranes can be determined using the density calculated from QCM-D and ellipsometry measurements:

$$\rho_{measured} = \frac{m_{LbL\ materials} + m_{water}}{A \cdot d} = \frac{m_{LbL\ materials} + \rho_{water} \cdot A \cdot (d - t_{nanosheet})}{A \cdot d_{calculated}}$$

where $\rho_{measured}$ is the density calculated from QCM-D and ellipsometry measurement in either dry or hydrated state; $m_{LbL\ materials}$ is the mass of the $MoS_2$ and PDDA/PrGO deposited; $m_{water}$ is the mass of water between stacked nanosheets; A is the area of the nanosheet; and $d_{calculated}$ is the interlayer spacing at either dry or hydrated state.

The $m_{LbL\ materials}$ per unit area (g-nm/cm$^2$) can be calculated from the density of $MoS_2$ and PDDA/PrGO, and the interlayer spacing measured by XRD at dry state:

$$\frac{m_{MoS2-PDDA}}{A} = (\rho_{MoS2} \cdot n_{MoS2} + \rho_{PDDA} \cdot n_{PDDA}) \cdot d_{XRD(MoS2-PDDA)}$$

Where $\rho_{MoS2}$ is 3.5 g/cm$^3$, $\rho_{PDDA}$ is 1.3 g/cm$^3$; $n_{MoS2}$ and $n_{PDDA}$ are the weight proportion of each material, which are 0.9 and 0.1 according to the QCM-D results; $d_{XRD}$ is the interlayer spacing of the $MoS_2$-PDDA membrane at dry state that measured by XRD (0.62 nm).

Similarly, the mass of $MoS_2$-PrGO per unit area can be calculated using:

$$\frac{m_{MoS2-PrGO}}{A} = (\rho_{MoS2} \cdot n_{MoS2} + \rho_{PrGO} \cdot n_{PrGO}) \cdot d_{XRD(MoS2-PrGO)}$$

$d_{XRD}$ is 0.93 nm measured by XRD.

The $m_{water}$ can be calculated from the density of water ($\rho_{water}$=1 g/cm$^3$) and the free spacing between stacked nanosheets. The free spacing is calculated by subtracting the thickness of one nanosheet ($t_{nanosheet}$=0.32 nm) from the interlayer spacing d.

The interlayer spacing can now be estimated, and the numbers are tabulated below:

| LbL membrane | $d_{XRD}$ (nm) | Mass per unit area (g-nm/cm$^2$) | $\rho_{measured}$ in dry state (g/cm$^3$) | $d_{calculated}$ in dry state (nm) | $\rho_{measured}$ in hydrated state (g/cm$^3$) | $d_{calculated}$ in hydrated state (nm) |
|---|---|---|---|---|---|---|
| $MoS_2$-PDDA | 0.62 | 2.084 | 3 | 0.88 | 2 | 1.76 |
| $MoS_2$-PrGO | 0.93 | 2.556 | 2.3 | 1.20 | 2.2 | 1.33 |

**Text S7. Calculating the advection coefficient**

The advection coefficient (α) of the membrane using the solution-diffusion imperfection relationship:

$$J_s = B\left(C_w - C_p\right) + \alpha J_w C_w$$

Where $J_s$ is the solute flux, calculated using $J_s$=$J_w$*$C_p$; B is the solute permeability coefficient; $C_w$ is the concentration of PEG in the feed (300 mg/L); $C_p$ is the concentration of PEG in the permeate. The equation can be re-written as:

$$1 - R = B\left(\frac{C_w - C_p}{J_w C_w}\right) + \alpha$$

Figure S14 shows the correlating between 1-R and $\frac{C_w - C_p}{J_w C_w}$. The value of the slope is the solute permeability coefficient, B, and the value of the y-intercept is the advection coefficient, α.

The solute flux from advection ($J_{s\text{-}adv}$) and diffusion ($J_{s\text{-}diff}$) can be calculated by:

$$J_{s-adv} = \alpha J_w C_w$$

$$J_{s-diff} = B\left(C_w - C_p\right)$$

Their proportion to total solute flux is:

$$Proportion\ of\ advective\ solute\ flux = \frac{J_{s-adv}}{J_s} \cdot 100\%$$

$$Proportion\ of\ diffusive\ solute\ flux = \frac{J_{s-diff}}{J_s} \cdot 100\%$$

**References**

(1) Zhao, C.; Lin, H.; Zhang, Q.; Na, H. Layer-by-Layer Self-Assembly of Polyaniline on Sulfonated Poly(Arylene Ether Ketone) Membrane with High Proton Conductivity and Low Methanol Crossover. *International Journal of Hydrogen Energy* **2010**, *35* (19), 10482–10488. https://doi.org/10.1016/j.ijhydene.2010.07.157.

(2) Zhou, J. Y.; Qin, Z. P.; Lu, Y. H.; Li, X. T.; An, Q. F.; Ji, S. L.; Wang, N. X.; Guo, H. X. MoS2/Polyelectrolytes Hybrid Nanofiltration (NF) Membranes with Enhanced Permselectivity. *J. Taiwan Inst. Chem. Eng.* **2018**, *84*, 196–202. https://doi.org/10.1016/j.jtice.2018.01.015.

(3) Lau, W.-J.; Ismail, A. F. Polymeric Nanofiltration Membranes for Textile Dye Wastewater Treatment: Preparation, Performance Evaluation, Transport Modelling, and Fouling Control — a Review. *Desalination* **2009**, *245* (1), 321–348. https://doi.org/10.1016/j.desal.2007.12.058.

(4) Fang, S.-Y.; Gong, J.-L.; Tang, L.; Cao, W.-C.; Li, J.; Tan, Z.-K.; Niu, Q.-Y.; Chen, Z.-P. Construction the Hierarchical Architecture of Molybdenum Disulfide/MOF Composite Membrane via Electrostatic Self-Assembly Strategy for Efficient Molecular Separation. *Chemical Engineering Journal* **2022**, *449*, 137808. https://doi.org/10.1016/j.cej.2022.137808.

(5) Liu, L.; Qu, S.; Yang, Z.; Chen, Y. Fractionation of Dye/NaCl Mixtures Using Loose Nanofiltration Membranes Based on the Incorporation of WS2 in Self-Assembled Layer-by-Layer Polymeric Electrolytes. *Ind. Eng. Chem. Res.* **2020**, *59* (40), 18160–18169. https://doi.org/10.1021/acs.iecr.0c03519.

(6) Tang, X.; Guo, B.; Zhang, S.; Tan, X.; Zheng, H. Layer-by-Layer Repaired Lamellar Membrane for Low Stacking Defect of MXene Nanosheets and Efficient Separation Performance in Water Purification. *Journal of Environmental Chemical Engineering* **2023**, *11* (2), 109450. https://doi.org/10.1016/j.jece.2023.109450.

(7) Sun, L.; Ying, Y.; Huang, H.; Song, Z.; Mao, Y.; Xu, Z.; Peng, X. Ultrafast Molecule Separation through Layered WS2 Nanosheet Membranes. *ACS Nano* **2014**, *8* (6), 6304–6311. https://doi.org/10.1021/nn501786m.

(8) Schneider, R.; Tandel, A. M.; Deng, E.; Correa, D. S.; Lin, H. Scalable Synthesis of Ultrathin MoS2 Membranes for Dye Desalination. *Journal of Membrane Science Letters* **2023**, *3* (2), 100058. https://doi.org/10.1016/j.memlet.2023.100058.

(9) Wang, Z.; Tu, Q.; Zheng, S.; Urban, J. J.; Li, S.; Mi, B. Understanding the Aqueous Stability and Filtration Capability of MoS2 Membranes. *Nano Letters* **2017**, *17* (12), 7289–7298. https://doi.org/10.1021/acs.nanolett.7b02804.

(10) Xiang, J.; Wang, X.; Ding, M.; Tang, X.; Zhang, S.; Zhang, X.; Xie, Z. The Role of Lateral Size of MXene Nanosheets in Membrane Filtration of Dyeing Wastewater: Membrane Characteristic and Performance. *Chemosphere* **2022**, *294*, 133728. https://doi.org/10.1016/j.chemosphere.2022.133728.

(11) Wang, H.; He, Z.; Yang, Q.; Zeng, G.; Yang, Z.; Pu, S. Fabrication of 2D/2D Composite Membrane via Combining Functionalized MXene and MoS2 Nanosheets for Dye/Salt Separation. *Journal of Environmental Chemical Engineering* **2022**, *10* (5), 108365. https://doi.org/10.1016/j.jece.2022.108365.

(12) PubChem. *Diallyldimethylammonium chloride.* https://pubchem.ncbi.nlm.nih.gov/compound/33286 (accessed 2023-10-08).
